# Effect of Oral Nutritional Supplementation on Growth in Children with Undernutrition: A Systematic Review and Meta-Analysis

**DOI:** 10.3390/nu13093036

**Published:** 2021-08-30

**Authors:** Zhiying Zhang, Fei Li, Bridget A. Hannon, Deborah S. Hustead, Marion M. Aw, Zhongyuan Liu, Khun Aik Chuah, Yen Ling Low, Dieu T. T. Huynh

**Affiliations:** 1 Abbott Nutrition Research and Development Asia-Pacific Center, 20 Biopolis Way, Unit 09-01/02 Centros Building, Singapore 138668, Singapore; zhiying.zhang@abbott.com (Z.Z.); zhongyuan.liu@abbott.com (Z.L.); khunaik.chuah@abbott.com (K.A.C.); 2 Abbott Nutrition China Research and Development Center, Building 14, No. 1036 Tianlin Road, Shanghai 200233, China; fei.li@abbott.com; 3 Abbott Nutrition Research and Development, 3300 Stelzer Road, Columbus, OH 43219, USA; bridget.hannon@abbott.com (B.A.H.); dhustead@insight.rr.com (D.S.H.); yenling.low@abbott.com (Y.L.L.); 4 Department of Paediatrics, Yong Loo Lin School of Medicine, National University of Singapore, Singapore 119077, Singapore; paeawm@nus.edu.sg

**Keywords:** malnutrition, undernutrition, children, oral nutritional supplements, meta-analysis, review

## Abstract

Oral nutritional supplements (ONS) are used to promote catch-up growth in children with undernutrition. We conducted a systematic review and meta-analysis to summarize the evidence of ONS intervention effects on growth for 9-month- to 12-year-old children who were undernourished or at nutritional risk. Eleven randomized controlled trials met the inclusion criteria; trials compared changes in anthropometric measures in children using ONS or ONS + DC (dietary counselling) to measures for those following usual diet or placebo or DC alone. The RCTs included 2287 children without chronic diseases (mean age 5.87 years [SD, 1.35]; 56% boys). At follow-up time points up to 6 months, results showed that children in the ONS intervention group had greater gains in weight (0.423 kg, [95% confidence interval 0.234, 0.613], *p* < 0.001) and height (0.417 cm [0.059, 0.776], *p* = 0.022) versus control; greater gains in weight (0.089 kg [0.049, 0.130], *p* < 0.001) were evident as early as 7–10 days. Longitudinal analyses with repeated measures at 30, 60, and 90 days showed greater gains in weight parameters from 30 days onwards (*p* < 0.001), a trend towards greater height gains at 90 days (*p* = 0.056), and significantly greater gains in height-for-age percentiles and Z-scores at 30 and 90 days, respectively (*p* < 0.05). Similar results were found in subgroup analyses of studies comparing ONS + DC to DC alone. For children with undernutrition, particularly those who were mildly and moderately undernourished, usage of ONS in a nutritional intervention resulted in significantly better growth outcomes when compared to control treatments (usual diet, placebo or DC alone).

## 1. Introduction

The World Health Organization (WHO) uses growth in children as an indicator of nutritional status. The number of children worldwide with poor growth today, despite a decline over the past 2 decades, remains high [1,2]. Growth statistics from 2019 showed that 144 million children under 5 years old were stunted, and 47 million were wasted [3]. Globally, 99 million children under 5 years old were underweight in 2013, while 75 million girls and 117 million boys aged 5–19 years old were moderately or severely underweight [3]. Undernutrition in different forms—evidenced by stunting, underweight, and wasting—is associated with increased morbidity and mortality from infections and other disease, in particular diarrhea and pneumonia [1,4,5]. Stunting in children is associated with not only loss of physical growth potential but also delayed motor and neurodevelopment, as well as impaired cognitive function [1,6,7,8,9,10,11,12]. If undernutrition and poor growth are not addressed, some consequences are irreversible and can negatively affect the ability of these children to reach their full productive potential as adults [3,6,13].

Undernutrition is a leading cause of growth restriction in children [14]. Growth faltering, in particular stunting, tends to occur in the first 1000 days between conception and a child’s second birthday [15,16]. Poor complementary feeding during this critical time has been identified as a risk factor associated directly with stunting [17]. Substantial accumulation of growth deficit was found to continue to age 5 years and can carry over to adulthood, and it may eventually become shorter as adults [16,18]. Evidence suggests that adequate nutrition and healthy growth in the first 1000 days of life contribute to long-term health benefits in later life [15]. Although administration of nutritional interventions to undernourished children can promote catch-up growth more effectively in the very young [19,20], catch-up growth in later childhood and adolescence can still occur with appropriate interventions [10,21]. Therefore, efforts to prevent further growth faltering and promote catch-up growth should also be given to children who have missed the first 1000 day window.

Close monitoring of child’s growth provides early identification of undernutrition, which warrants timely nutrition intervention to prevent growth faltering. Many clinical studies have shown that administration of nutritional supplements to undernourished children can lead to improved nutrient intake which drives catch-up growth and better growth parameters [22,23,24]. Based on the WHO guidelines for catch-up growth, such nutritional management should prioritize the provision of sufficient energy and protein to support catch-up growth [6].

Children with growth shortfalls need nutritional interventions aimed at restoring growth to normal patterns in a timely manner. Importantly, today’s health care professionals aim to identify children when they begin to show evidence of poor growth, thus facilitating much earlier nutritional interventions. To this end, WHO has developed a full set of childhood growth standards by age and sex [25]. The three most commonly used anthropometric indices are weight-for-age, height-for-age, and weight-for-height, which can be expressed as Z-scores (standard deviation scores) and percentiles [25,26]. Other measures, including body mass index (BMI)-for-age and mid-arm circumference-for-age, are also used to assess growth [25]. The risk of undernutrition is statistically defined as a Z-score between −1 and −2, which indicates the child has evidence of mildly poor growth and is at risk for undernutrition. A Z-score between −2 and −3 indicates moderate undernutrition, and <−3 signifies severe undernutrition [27]. Stunting is defined as height-for-age Z-score (HAZ) < −2, wasting as weight-for-height Z-score (WHZ) < −2, and underweight as weight-for-age Z-score (WAZ) < −2.

For children in whom conditions of poor growth and undernutrition have been identified, there is an urgent need to build knowledge on the most impactful strategies for treatment, especially because some consequences of undernutrition can have lasting negative effects throughout their lives [3,6,13]. The treatment goal is for each child to consume a diet containing adequate and balanced nutrition; both from the point of macronutrients and micronutrients. This reduces the risk of poor linear growth and underweight [28]. Children in some populations may not get sufficient nutrient intake as a result of caregiver’s poor knowledge and lack of awareness on the importance of nutrition, economic constraints, lack of access, or cultural preferences [29]. At the same time, underlying poor sanitation and hygiene problems result in increased infections which further impair growth and must be addressed [30,31]. Various types of dietary supplementation have been explored as ways to help meet nutritional and growth goals for children with growth shortfalls. Systematic reviews and meta-analyses have shown that certain dietary supplements have some growth benefits, but evidence is currently limited to micronutrient or protein supplementation with milk and other animal-sourced foods [32,33,34,35]. Such findings suggest that nutritional interventions with both macronutrients and essential micronutrients are needed to restore growth to normal in at-risk or undernourished children.

This systematic review and meta-analysis aimed to summarize evidence regarding the effects of oral nutritional supplements (ONS) on catch-up growth in 9-month- to 12-year-old children with undernutrition. Our study helps fill an important knowledge gap because no other systematic review has yet been conducted to specifically investigate the impact of ONS on growth in children with undernutrition or its risk.

## 2. Materials and Methods

### 2.1. Conduct of Review 

This review was planned and conducted following the Cochrane Handbook for Systematic Reviews of Interventions [36]. The protocol was registered in PROSPERO with study number CRD42017070623. The results were reported in accordance with the Preferred Reporting Items for Systematic Reviews and Meta-Analyses (PRISMA) guideline [37].

### 2.2. Search Strategy

We searched Cochrane, ProQuest Dialog (PQD), Scopus, and other relevant sources for randomized controlled trials (RCTs) investigating the effects of ONS on growth among children below 18 years of age who had varying degrees of undernutrition as indicated by anthropomorphic measures, and who did not have chronic diseases. Our search strategy included the following keywords: (oral nutrition* supplement*) AND (weight OR height OR growth) AND (malnutrition OR malnourish* OR undernutrition OR undernourish*). The detailed search strategy for PQD can be found in Supplementary Table S1. The bibliographic references of all selected studies and review articles were manually screened for additional eligible studies. An attempt to obtain additional data was also made by direct contact with authors in the field of interest. The last search was run on 12 April 2021.

### 2.3. Inclusion Criteria

We included randomized or quasi-randomized trials of children ages below 18 years with a mild, moderate, or severe degree of undernutrition. In order to be included, trials must have evaluated one of the following: (i) ONS compared with placebo (low-nutrient ONS); (ii) ONS + DC compared with DC; or (iii) ONS compared with habitual diet. Studies of children with chronic diseases, such as cystic fibrosis, HIV/AIDS, or malignancies were excluded, as the effects of ONS on these populations were recently reviewed [38,39]. We included studies that had been conducted with children who were otherwise considered clinically healthy, or who had acute infections of the respiratory and gastrointestinal tracts but did not require hospitalization.

Pediatric ONS are designed to be consumed orally by children who do not get adequate nutrition by food intake alone. They are liquid, semi-solid or powder formulas containing at least one non-protein source of calories (carbohydrate and/or fat) and nitrogen (as intact protein, digested protein, and/or amino acids) in balanced amounts, as well as a wide range of micronutrients to supplement or use as the sole source to provide complete nutritional requirements for an individual. Dietary counselling is defined as instructions to modify food intake, which is usually provided to help individuals improve their nutritional intake when they make and maintain the needed dietary changes.

We focused on studies using cow-milk-based polymeric ONS, as these are suitable for most pediatric patients [40]. Studies of other types of formula, such as those with pre-digested nutrients, were excluded from this review. Fortified blended foods, such as corn-soy or wheat-soy flours (with or without sugar and oil), and micronutrient-only supplements or powders were also excluded. Ready-to-use therapeutic food (RUTF) was excluded, as it has been recently reviewed by Fatima et al. [41]. Studies on nutritional supplements that contain only or predominantly one macronutrient, such as lipid-based nutrient supplements (LNS), were not eligible for inclusion because several ongoing systematic reviews on LNP for childhood malnutrition have been registered [42,43].

### 2.4. Anthropometric Measures 

When providing a nutritional intervention for undernourished or at-risk children, the goal is to enable catch-up growth in weight and height. Outcomes considered for this review were changes in height (centimeters), weight (kilograms), and body mass index (kilograms/meters^2^), and the age- and sex- specific Z-scores and percentiles for these parameters [44]. These included weight-for-age Z-score (WAZ) or weight-for-age percentile (WAP), height-for-age Z-score (HAZ) or height-for-age percentile (HAP), weight-for-height Z-score (WHZ) or weight-for-height percentile (WHP), BMI for age Z-score (BMIAZ) or BMI for age percentile (BMIAP), and mid-upper-arm circumference (MAC, cm) [26].

### 2.5. Nutritional Intake 

Nutritional intervention using ONS aims to increase energy and nutrient intake. In this review, change in total energy intake (kcal) before and after the intervention was considered. ONS provides both macro- and micronutrients. Therefore, energy intake can be used as a proxy for nutrient intake. 

### 2.6. Study Selection and Data Extraction

Titles and abstracts retrieved from the databases were screened independently by two reviewers to identify relevant studies meeting the selection criteria outlined above, who also independently assessed the eligibility by further reviewing the full text. Any disagreement was resolved by consultation with a third reviewer. 

Two reviewers extracted data independently and discrepancies were identified and resolved through consultation with a third reviewer. A standardized, pre-piloted data collection table was used to extract data from the included studies using the Systematic Review Data Repository [45]. The primary outcome required was the mean difference and standard deviation (SD) of the changes in anthropometric measures between the intervention group and the comparison group. 

We requested raw data from study authors for missing data and for clarifying values of measurements. Where appropriate, the missing mean changes and SDs were estimated following the Cochrane Handbook [36]. Some studies reported changes in either percentile or Z-scores but not both, thus percentiles and Z-scores were inter-converted based on their 95% confidence intervals (CIs). 

### 2.7. Study Quality Assessment 

We used the Grading of Recommendations, Assessment, Development, and Evaluation (GRADE) guideline (http://www.gradeworkinggroup.org/, accessed on 11 July 2020) to evaluate the overall quality of evidence for each outcome. The GRADE assessment employed the following criteria: risk of bias and study limitations, directness, consistency of results, precision, publication bias, magnitude of effect, dose–response gradient, and residual confounding. Risk of bias was assessed using the Cochrane risk of bias (ROB) assessment tool, based on criteria described by Schulz et al. [46], as specified in the Cochrane Handbook for Systematic Reviews of Interventions [36]. Two reviewers independently assessed the ROB and GRADE quality of each study. The GRADE quality was subsequently rated as “high”, “moderate”, “low”, or “very low”. Any disagreements between the reviewers were resolved by the third reviewer.

### 2.8. Statistical Analysis

For continuous variables, we recorded the mean change from baseline and the SD for each group. Studies using standard errors instead of SDs were converted to SDs. Estimates of the treatment effect were created by calculating the mean difference (MD) and the corresponding 95% CI. 

All analyses were conducted using the change from baseline for each group, and the mean difference between groups was calculated as a comparison between the ONS treatment group and the non-ONS group (control) for each study. The difference in mean change from baseline was examined in a univariate fashion for the longest follow-up time point reported in the study and for each reported length of intervention separately. When possible, we conducted longitudinal analyses for studies with repeated measures at 30, 60, and 90 days.

We performed meta-analyses when more than two studies were identified for each outcome; not all studies were included in each analysis. A narrative summary was provided for studies that could not be included in meta-analyses. Continuous outcomes, such as change in weight, were combined across studies using a mean difference and 95% CI. The heterogeneity among studies was estimated by Q test and I^2^ statistic. A *p*-value < 0.1 and I^2^ over 50% indicate a high level of heterogeneity [36]. There were between-study differences in the ages of children included in the analyses, the health status or underlying condition of the children, the placebo/control feedings used, and the specific type of ONS intervention. All these factors may have had an impact on the observed effect size. It was therefore determined a priori that analyses would be conducted using a random-effects model.

We assessed publication bias by visual inspection of the funnel plot asymmetry and using Egger’s test. Due to the relatively small number of studies used, these need to be interpreted with caution.

A sensitivity analysis was conducted with and without the studies assessed as potentially having a high risk of bias and low quality. Subgroup analyses were conducted to determine if ONS is more effective for studies using consistent study designs, e.g., comparing ONS + DC vs. DC alone. Two-sided *p*-value was used with an α level of 0.05. Meta-analyses were performed using the software Comprehensive Meta-Analysis, version 3.0 (Biostat, Inc., Englewood, NJ, USA).

## 3. Results

### 3.1. Study Characteristics

**Figure 1:**
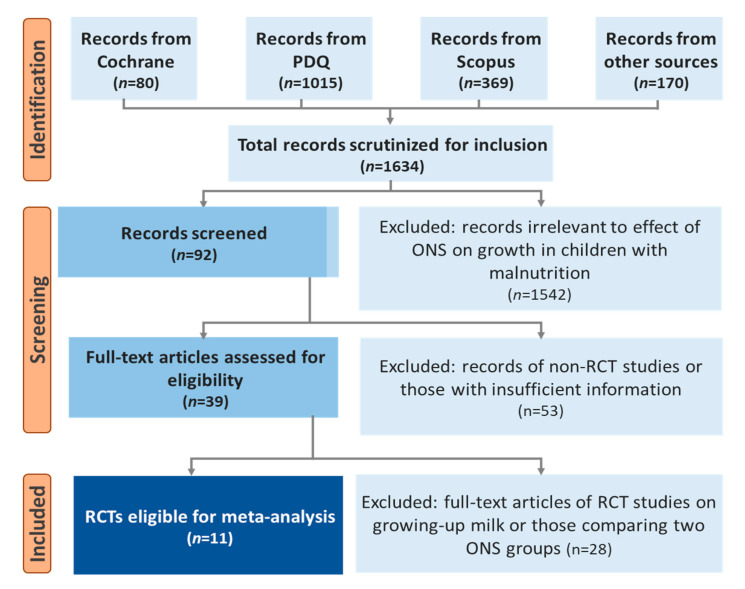
Identification, screening, and selection of articles for the meta-analysis.

Eleven studies met the inclusion criteria (Figure 1). Of these, seven were considered to be of “high” quality [23,47,48,49,50,51,52] and two each “moderate” [53,54] and “low” [55,56] quality (Table 1). 

The RCTs (*n* = 11, Table 1) represented 2287 children without chronic diseases (mean age in years 5.87, SD 1.35; 56% boys), most with mild-to-moderate undernutrition. One of the RCTs (Cervo [53]) was designed to include both normal and underweight children. However, only the results for underweight and severely underweight children were used for meta-analysis. Two of the RCTs (Alarcon [23], Sheng [49]) included some normal children with an anthropometric measure above the 15.9th percentile but below the 25th percentile; these were also considered. A majority of the subjects were from less developed countries. The number of children included in a single trial ranged from 20 to 842, and the length of the interventions ranged from 7 to 365 days. There were only three studies [48,54,56] with reported outcomes at 365 days of intervention, one of which was rated to be of low quality [56]. Therefore, meta-analyses were only performed for the outcomes of interest from 7 to 180 days. This precluded the study by Vijayalakshmi et al. [56] from being used in any meta-analysis due to only reporting data at 365 days. For the study by Cervo et al. [53], the intervention period was 84 days, the results of which were pooled with other studies with 90 days of intervention. No RCTs were available for children above 12 years old. The meta-analysis thus included studies of young children and preadolescent children.

Eight RCTs reported outcomes within a period of 30–90 days (Alarcon [23], Han [48], Sheng [49], Lebenthal [51], O’Reilly [55], Cervo [53], Ghosh [50], Khanna [57]). Four of those RCTs reported results for multiple intervention time points including 30, 60, and 90 days (Alarcon [23], Khanna [57], Ghosh [50], and Sheng [49]). These four RCTs consistently covered children 2–6 years old and were all designed to compare ONS + DC vs. DC alone. Different types of interventions were used in the remaining studies, including (1) usual diet + nutrient-fortified milk-based formula vs. usual diet (Cervo [53]); (2) a nutritional supplement with or without synbiotics vs. a non-ONS fruit-flavored drink (Schrezenmeir [47]); (3) daily nutritional supplementation vs. observation (Han [48]); (4) a milk-based supplement providing the total protein requirement vs. home diet, both study arms with or without psychosocial stimulation (Walker [54]); (5) a sachet of a nutritional supplementation formula to be combined with water vs. a placebo supplement (Lebenthal [51] and Rawat [58]). For studies with multiple ONS arms (Schrezenmeir [47] and Khanna [57]), the outcomes for the different ONS intervention groups were pooled and then compare to the control group.

### 3.2. Quality of Included Studies and Risk of Bias

The quality of the included studies, as assessed by GRADE, is shown in Table 1. Most of the studies were graded as high quality, except for two each that were graded as poor (Vijayalakshmi [56] and O’Reilly [55]) or moderate quality (Walker [54] and Cervo [53]). 

The risk of bias of included studies is presented in Supplementary Figure S1. For the risk of bias assessment, two papers (Lebenthal [51] and Yackobovitch-Gavan [59]) and one poster (Rawat [58]) were assessed as one study since they all utilized the same protocol and subjects. All studies were at low risk for reporting bias. Two studies (Alarcon [23] and Han [48]) were at high risk of detection bias because the outcome assessors were not blinded to the treatment groups. Three studies (Lebenthal [51], Sheng [49], and Cervo [53]) had a higher risk for attrition bias due to higher drop-out rates. Seven studies (Walker [54], Schrezenmier [47], Han [48], O’Reilly [55], Cervo [53], Ghosh [50], and Vijayalakshmi [56]) were at high risk of performance bias for subjects, researchers, or both, for being unblinded to treatment group assignment.

### 3.3. Results for Weight Parameters: Change in Weight, Weight-for-Age, and Weight-for-Height

**Figure 2:**
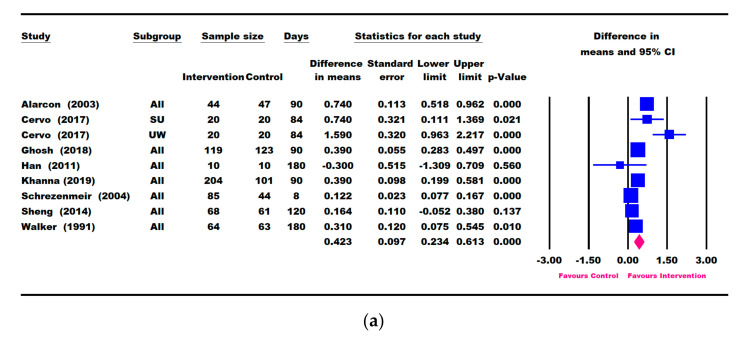

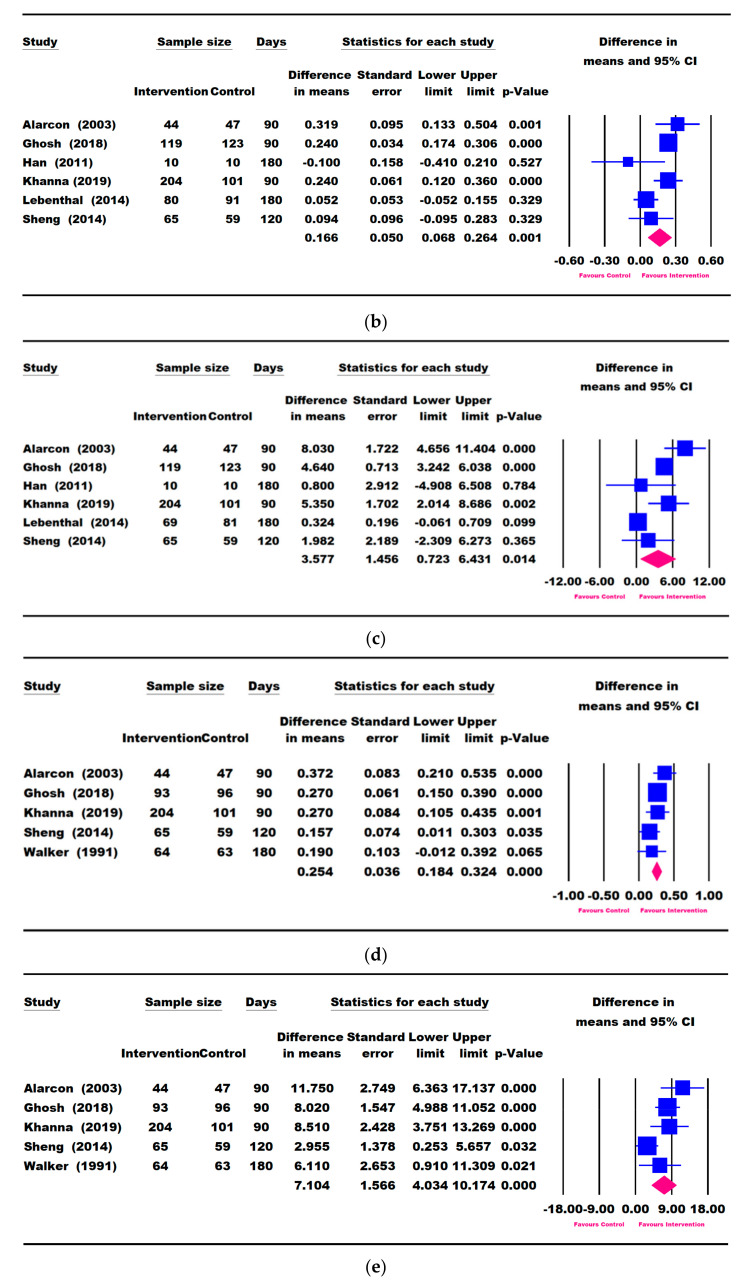
Meta-analysis results on the difference in mean change in weight parameters between intervention and control based on the longest follow-up time point. (**a**) Weight (kg), (**b**) weight-for-age Z-score (WAZ), (**c**) weight-for-age percentile (WAP), (**d**) weight-for-height Z-score (WHZ), (**e**) weight-for-height percentile (WHP). SU: severely underweight; UW: underweight. The forest plot shows the mean difference (squares) and 95% confidence intervals (CIs) (horizontal bars) for intervention vs. control. The values were combined using a meta-analysis to obtain a pooled estimate of the effect from all the included studies (diamond).

Eight studies (Alarcon [23], Cervo [53], Khanna [57], Ghosh [50], Han [48], Schrezenmeir [47], Sheng [49], and Walker [54]) reported outcomes in weight gain (“catch-up weight”) from baseline (Figure 2a). 

Six and five studies, respectively, reported changes in weight-for-age and weight-for-height data (Figure 2b–e). A meta-analysis based on the longest follow-up time point (up to 6 months) showed that children in the ONS group had significantly greater catch-up growth for weight (0.423 kg, 95% CI [0.234, 0.613] kg, *p* < 0.001), WAZ (0.166 [0.068, 0.264], *p* = 0.001), WAP (3.577 [0.723, 6.431], *p* = 0. 014), WHZ (0.254 [0.184, 0.324], *p* < 0.001), and WHP (7.104 [4.034, 10.174], *p* < 0.001) compared with the control group (Figure 2).

Three studies (Khanna [57], Ghosh [50], and Schrezenmeir [47]) reported change in weight within 7–10 days of ONS intervention. A significantly greater weight gain (0.089 [0.049, 0.130] kg, *p* < 0.001) was observed in 7–10 days of intervention in the ONS group compared with the control group Supplementary Figure S2).

**Figure 3:**
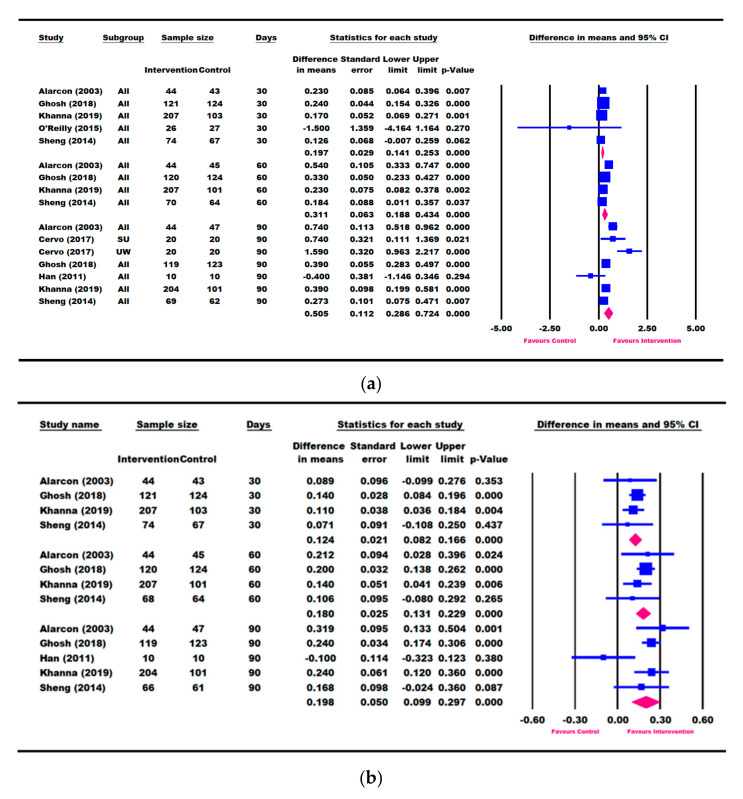

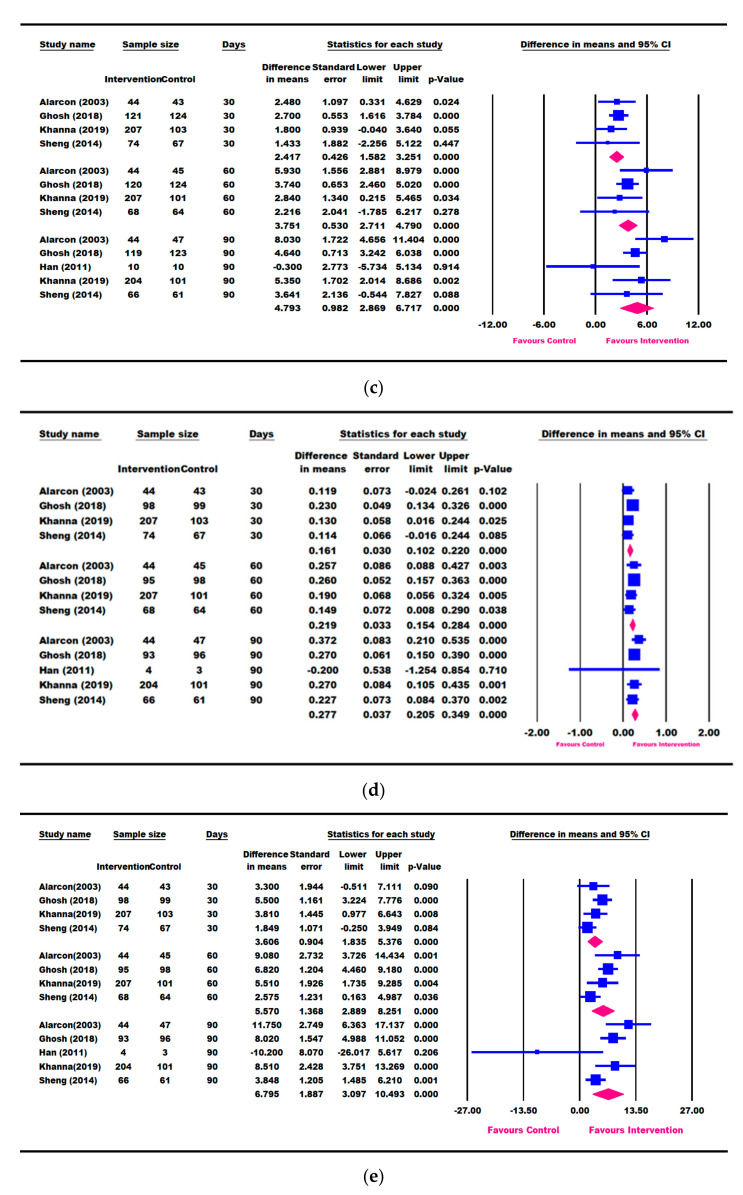
Meta-analysis results on the difference in mean change in weight parameters between intervention and control after 30, 60, and 90 days of intervention. (**a**) Weight (kg), (**b**) weight-for-age Z-score (WAZ), (**c**) weight-for-age percentile (WAP), (**d**) weight-for-height Z-score (WHZ), (**e**) weight-for-height percentile (WHP). SU: severely underweight; UW: underweight. The forest plot shows the mean difference (squares) and 95% confidence intervals (CIs) (horizontal bars) for intervention vs. control. The values were combined using a meta-analysis to obtain a pooled estimate of the effect from all the included studies (diamond).

Seven studies (Alarcon [23], O’Reilly [55], Cervo [53], Khanna [57], Ghosh [50], Han [48], and Sheng [49]) reported change in weight within a period of 30–90 days of ONS intervention (Figure 3). Results showed that children in the ONS intervention group continued to gain more weight from 0.197 kg (95% CI [0.141, 0.253] kg, *p* < 0.001) at 30 days to 0.505 kg (95% CI [0.286, 0.724] kg, *p* < 0.001) at 90 days, compared with the control group. A sensitivity analysis that removed the O’Reilly (2015) study [55] that was deemed to be of low quality did not alter the results in mean weight change at 30 days (data not shown). 

Four RCTs (Alarcon [23], Khanna [57], Ghosh [50], and Sheng [49]) consistently intervened with ONS + DC compared to DC alone. These four studies also had repeated weight, weight-for-age and weight-for-height measures at 30, 60, and 90 days. We therefore conducted a meta-analysis based on these four RCTs (Supplementary Figure S3). Children receiving ONS + DC for 30 days had significantly greater improvements in weight (0.198 [0.143, 0. 0.253] kg, *p* < 0.001), WAZ (0.124 [0.082, 0.166], *p* < 0.001), WAP (2.417 [1.582, 3.251], *p* < 0.001), WHZ (0.161 [0.102, 0.220], *p* < 0.001), and WHP (3.606 [1.835, 5.376], *p* < 0.001) than those receiving DC alone. Children receiving ONS + DC continued to show significantly greater weight gain, WAZ, WAP, WHZ, and WHP at 60 and 90 days compared with DC alone (Supplementary Figure S3). 

There was only one study that reported results at 120 days (Sheng [49]). As for results at 180 days, two studies each reported change in weight (Han [48] and Walker [54]) and weight-for-age (Han [48] and Lebenthal [51]), with only one study for weight-for-height (Walker [54]). Therefore, a meta-analysis was not performed for these time points.

### 3.4. Results for Height Parameters: Change in Height and Height-for-Age

**Figure 4:**
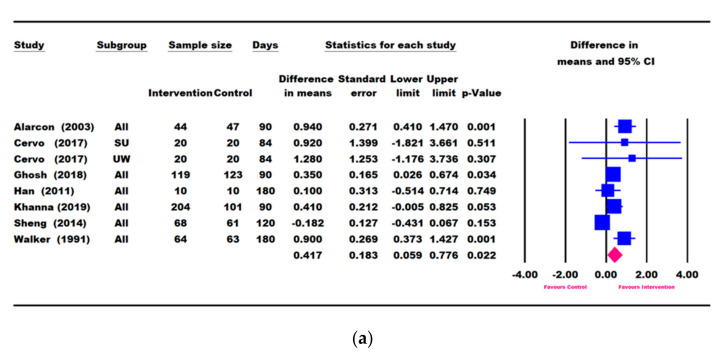

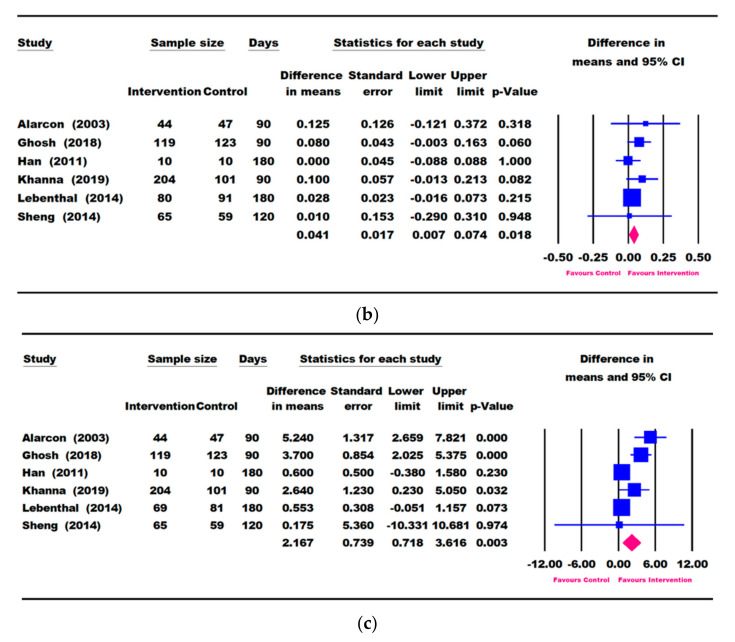
Meta-analysis results on the difference in mean change in height parameters between intervention and control based on the longest follow-up time point. (**a**) Height (cm), (**b**) height-for-age Z-score (HAZ), (**c**) height-for-age percentile (HAP). SU: severely underweight; UW: underweight. The forest plot shows the mean difference (squares) and 95% confidence intervals (CIs) (horizontal bars) for intervention vs. control. The values were combined using a meta-analysis to obtain a pooled estimate of the effect from all the included studies (diamond).

Seven studies (Alarcon [23], Cervo [53], Khanna [57], Ghosh [50], Han [48], Sheng [49], and Walker [54]) reported change in height (cm) (Figure 4a), and all except Cervo [53] reported changes in HAZ and HAP (Figure 4b,c). A meta-analysis using the longest follow-up time point showed that children in the ONS group had significantly greater catch-up growth in height (0.417 [0.059, 0.776] cm, *p* = 0.022), HAZ (0.041 [0.007, 0.074], *p* = 0.018), and HAP (2.167 [0.718, 3.616], *p* = 0.003) compared with the control group (Figure 4).

**Figure 5:**
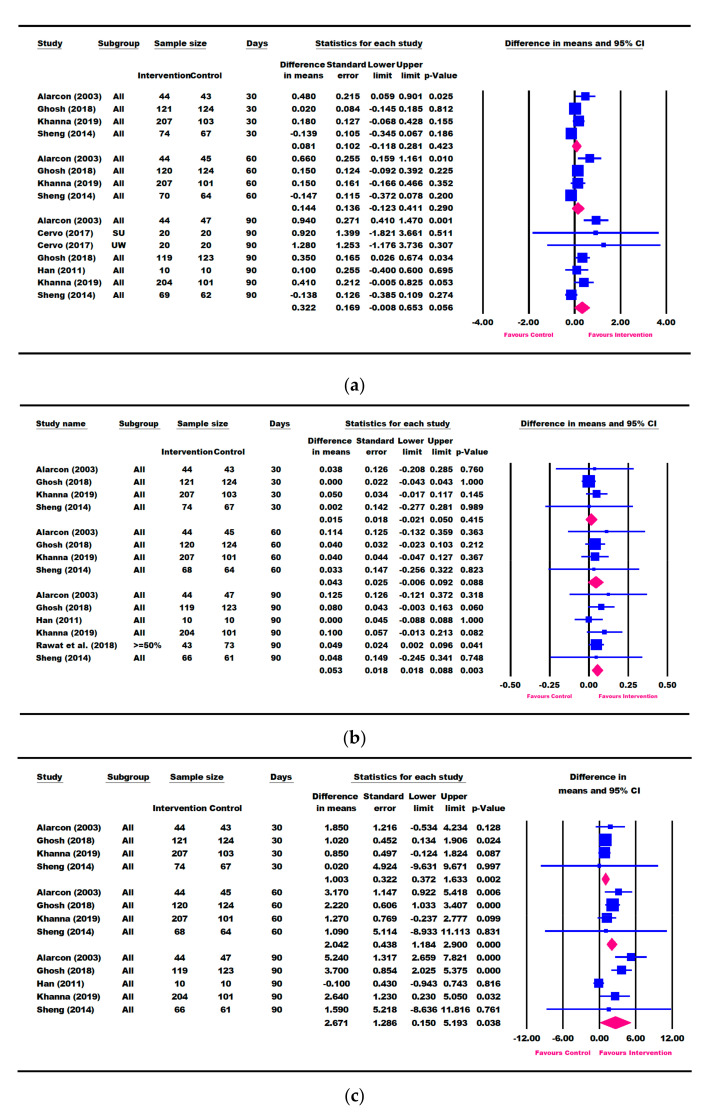
Meta-analysis results on the difference in mean change in height parameters between intervention and control after 30, 60, and 90 days of intervention. (**a**) Height (cm), (**b**) height-for-age Z-score (HAZ), (**c**) height -for-age percentile (HAP). SU: severely underweight; UW: underweight. The forest plot shows the mean difference (squares) and 95% confidence intervals (CIs) (horizontal bars) for intervention vs. control. The values were combined using a meta-analysis to obtain a pooled estimate of the effect from all the included studies (diamond).

Five RCTs (Alarcon [23], Khanna [57], Ghosh [50], Han [48], and Sheng [49]), along with Cervo [53] on height (cm) and Rawat [58] on HAZ, reported change in height parameters within a period of 30–90 days of ONS intervention (Figure 5). When compared with the control, the ONS group trended towards a greater catch-up in height in 90 days (0.322 cm [−0.008, 0.653], *p* = 0.056) and had significantly greater gains in HAP in 30 days (1.003 [0.372, 1.633], *p* = 0.002) as well as at 60 and 90 days and HAZ in 90 days (0.053 [0.018, 0.088], *p* = 0.003). A sensitivity analysis removing one study (Rawat [58]), which reported HAZ outcome for children with ≥ 50% compliance, did not alter the results for change in HAZ at 90 days (data not shown). 

Given that four out of these six RCTs consistently compared ONS + DC to DC alone and had repeated height, HAZ, and HAP measures at 30, 60, and 90 days of follow-up, we conducted a subgroup analysis. Meta-analysis results are shown in Supplementary Figure S4. There was a trend for a greater height gain in the intervention than in the control from 30 to 90 days, with the largest difference observed at 90 days (0.350 cm [−0.072, 0.772], *p* = 0.104). When compared with DC alone, the ONS + DC group reached a significantly greater HAZ gain at 90 days (0.088 [0.025, 0.151], *p* = 0.006) and achieved a significantly greater HAP gain at 30 days (1.003 [0.372, 1.633], *p* = 0.002). Three out of 4 RCTs reported a faster height gain in the ONS + DC group, varying from 36% (Ghosh [50]), 40% (Khanna [57]) and 55% (Alarcon [23]) (Supplementary Table S2). There was one study (Sheng [49]) that showed no significant difference in height gain between the intervention and control group.

There was only one study that reported results at 120 days (Sheng [49]). Two studies each reported change in height (Han [48] and Walker [54]) and height-for-age (Han [48] and Lebenthal [51]) at 180 days of intervention. Therefore, a meta-analysis was not performed for these time points.

### 3.5. Other Growth Outcomes

Children who received ONS also showed improvements in MAC, BMI, and BMIAZ at 90 days of ONS treatment, when compared with those receiving placebo/control (Supplementary Figure S5).

### 3.6. Nutritional Intake

**Figure 6:**
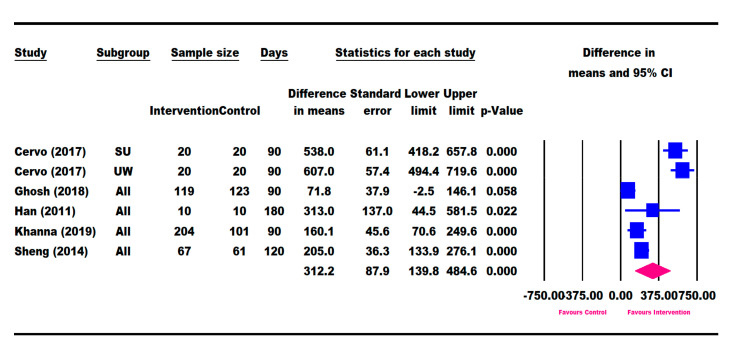
Meta-analysis results on the difference in change in total energy intake between intervention and control based on the longest follow-up time point. SU: severely underweight; UW: underweight. The forest plot shows the mean difference (squares) and 95% confidence intervals (CIs) (horizontal bars) for intervention vs. control. The values were combined using a meta-analysis to obtain a pooled estimate of the effect from all the included studies (diamond).

Out of the 11 selected RCTs, 8 RCTs (Cervo [53], Khanna [57], Ghosh [50], Han [48], Sheng [49], Schrezenmeir [47], Lebenthal [51], and Vijayalakshmi [56]) reported a significantly greater increase in total energy intake for the ONS intervention group compared to the control (Supplementary Figure S6), while the remaining three (Alarcon [23], O’Reilly [55], and Walker [54]) did not report total energy intake. Five RCTs (Cervo [53], Khanna [57], Ghosh [50], Han [48], and Sheng [49]) had data on the change in total energy intake from baseline to the end of the intervention. A meta-analysis of these five studies using the longest follow-up time point showed that the ONS group had a significantly higher increase in total energy intake (312.2 kcal [139.8, 484.6], *p* = 0.000) compared with the control group (Figure 6). Because the information for total energy intake was available for less than 3 RCTs for day 30, we did not conduct the longitudinal data analysis with repeated measures at 30, 60, and 90 days. For RCTs comparing ONS + DC versus DC, the energy intake information was available in three studies (Khanna [57], Ghosh [50], and Sheng [49]). However, because the intervention durations were different among these three studies, a subgroup analysis of the RCTs comparing ONS + DC versus DC alone was not conducted. 

### 3.7. Publication Bias and Heterogeneity

There was evidence of substantial heterogeneity (*p* < 0.001, I^2^ > 50%) across studies based on outcomes at the longest time point, partly because the outcomes were measured at various time points. The funnel plots for Z-score measures appeared to be symmetric, though funnel plots for weight and height (Supplementary Figure S7) suggested some publication bias. Estimation based on Duval and Tweedie’s Trim and Fill method [53] did not alter the results, however. Results need to be interpreted with caution due to the relatively small number of available studies.

## 4. Discussion

Millions of children in developing countries today still experience growth stunting, underweight, and wasting due to severe nutritional inadequacy [1,2,3]. At the same time, many other children worldwide though experiencing less-severe undernutrition still demonstrate negative effects on growth and health outcomes [60]. Interventions in the form of ONS offer the advantage of providing additional calories as well as important macro- and essential micronutrients to enable catch-up growth. 

In our systematic review and meta-analyses of ONS intervention studies for children with undernutrition or at nutritional risk, we found that the provision of ONS had significant positive effects on weight gain and height growth. The analysis using the longest follow-up time point showed that an intervention providing ONS resulted in a higher increase in energy intake and greater weight and height gains for undernourished or at-risk children when compared with the control groups receiving DC alone or a placebo control or usual diet. Analysis of the studies with repeated measures at 30, 60 and 90 days allowed for a comparison of the magnitude of changes at these different time points. The difference in mean change between the intervention and control at 90 days was significantly larger than that at 30 days for weight, WAZ, WAP, WHZ and HAP, indicating that the catch-up growth was increasingly greater in the intervention than control over time during the period of 90 days. Subgroup analyses including studies comparing ONS + DC to DC alone showed that children who received ONS + DC had significantly greater gains in weight, WAP, WAZ, WHP, WHZ, and HAP at 30, 60, and 90 days compared to children receiving DC alone. It is not surprising that the gains in height were seen later than the gains in weight. This suggests that nutritional supplementation in undernourished children should be given for sufficiently long for catch-up height to occur, and that this period is likely at least 90 days. In addition, children who received ONS also showed improvements in MUAC, BMI, and BMIAZ at 90 days. 

In this meta-analysis, the ONS intervention group had a significant increase in energy intake which was associated with a greater gain in weight and height when compared with the control receiving DC or a placebo control or usual diet. The control-group children who received DC alone reported gains in weight or height, although the effects were significantly smaller than those of the intervention group. Providing DC remains the first line of treatment to promote catch-up growth in nutritionally at-risk children. Studies comparing the effects of intervention with DC to control without DC, however, showed varying results from little to significant success [61,62]. Reasons for variability in results include the intensity of the dietary counselling, the behaviors to be changed, caregivers’ time constraints, and challenges in procuring dietary diversity to meet nutritional requirements [62]. Nutritional supplementation and food fortification are therefore recommended for achieving the desired nutrient density and nutrient adequacy to promote growth in children with undernutrition [63]. This review shows that DC using family foods is more effective when combined with ONS in promoting growth in children with undernutrition or nutritional risk, especially catch-up growth in weight and height over an intervention period up to 90 days. 

Roberts et al. [34] conducted a systematic review and meta-analysis of studies on specific dietary ingredients and linear growth for children over 2 years of age who were undernourished or at risk of undernutrition. According to the analyses, interventions providing iron, calcium, or iodine or those supplying foods did not improve linear growth, but interventions providing zinc, vitamin A, multiple micronutrients, or protein had positive effects on height. Intervention duration ranged from 6 to 24 months, and the dosage of micronutrients varied from the daily requirement to 6–8-fold higher than the daily recommendation for healthy children. It is also worth mentioning that single-nutrient or multiple-micronutrient supplementation without additional calories and macronutrients did not always promote catch-up weight in these trials. On the contrary, ONS supplementation providing a complete blend of macronutrients and micronutrients has been consistently shown to promote catch-up weight in children at nutritional risk. While growth faltering may stem from deficiencies in single micro- or macronutrients, poor growth is more commonly due to deficiencies of multiple nutrients in developing countries [64]. Therefore, ONS would be considered an effective nutrition intervention approach to tackle growth faltering in both weight and height compared with single-nutrient supplementation in at-risk or undernourished children. 

A recent Cochrane review by Das et al. [32] showed that LNS given jointly with complementary feeding as a preventive approach in vulnerable populations reduced stunting, underweight, and wasting in infants and young children (6–23 months). LNS contains macronutrients with fat as a major constituent and micronutrients. Despite the differences in terms of intervention strategies, study populations, and intervention duration, because multiple nutrient deficiencies are common in these children at risk of undernutrition, the findings from the review by Das et al. [32] and our study support the use of a nutritional supplement to provide macronutrients and micronutrients to help meet nutritional needs and improve nutritional status in children at risk of undernutrition.

While faltering growth is complex and often multifactorial, it is often due to inadequate nutritional intake, poor absorption, and ineffective utilization of nutrients [65]. When these underlying causes of undernutrition are solved, spontaneous catch-up growth usually occurs, bringing the child back to its original growth trajectory [14]. Complete or near-complete catch-up growth is possible in infants and young children if intervened early [66,67]. However, catch-up growth may be incomplete in children near or in puberty if the growth disorder carries over years due to late intervention [66,67]. In the present review, the included studies involving children aged 9 months to 12 years, yielding meta-analysis results covering young children and prepubertal children. No RCTs were available for children above 12 years old. Nevertheless, the evidence suggests catch-up growth occurs not only in early childhood but also in puberty [18,68]. Further studies are needed to evaluate the effect of ONS on promoting catch-up growth for children above 12 years old, particularly children during puberty.

### Study Strengths and Limitations

A major strength of our study is that it is the first systematic review to evaluate the effectiveness of ONS compared with a control group of usual diet, standard care (dietary counselling alone), or placebo on growth parameters in undernourished or at-risk children aged 9 months to 12 years. We sought to conduct a high-quality study by following published guidelines for such analyses, strictly adhering to the recommendations by the Cochrane Collaboration on intervention studies [53]. 

A limitation was the heterogeneity of studies, including a wide array of countries from which data were drawn and a broad range of publication dates. There were also differences in the age ranges of the children, the ONS and other supplemental formulations, durations of the interventions, and dosing and compliance (this information was not available for all studies). Conversion of Z-score to percentile (and vice versa) using a conservative correlation coefficient has resulted in a large SD and a wide 95% CI. Nonetheless, this is likely to underestimate rather than overestimate the treatment effects. Our final analyses represented a small number of studies with a relatively short duration of follow-up (approximately 90 days for most studies included). It would be important to determine if catch-up growth is sustained in the absence of ongoing ONS consumption. Longer follow-up periods may provide additional insight into the benefits and/or risks of supplementation.

## 5. Conclusions

The results of our review and meta-analysis showed that ONS intervention is effective in promoting better growth outcomes for children with undernutrition, particularly for children with a mild-to-moderate degree of undernutrition. Subgroup analyses showed that ONS + DC resulted in significantly greater gains in weight, WHZ, WHP, WAP, HAP, and WAZ at 30, 60, and 90 days in undernourished or at-risk children when compared to DC alone. Our findings are important because improved nutritional status for pre-schoolers and school-aged children positively impacts motor and cognitive development in youth, which in turn improves each child’s potential for a healthier and more productive adult life [8,9]. 

## Figures and Tables

**Table 1:** List of RCTs that met the eligibility criteria.

Study	Country	Subjects *	*n*	Age Range	Mean Age, Years (SD)	% Boys	Intervention Type	Intervention Intervals, Days	GRADE Quality
Walker, 1991	Jamaica	HAZ < −2 WHZ ≤ 0	127	9–24 m	1.56 (0.34)	56	ONS vs. no ONS	180, 365	Moderate
Alarcon, 2003	Philippines, Taiwan	WHP < 25th percentile, picky eater	104	3–5 y	4.04 (0.54)	51	ONS-DC vs. DC	30, 60, 90	High
Schrezenmeir, 2004	Germany	Acute infection	129	1–6 y	4.16 (1.48)	56	ONS vs. fruit-flavored drink	8	High
Vijayalakshmi, 2008	India	Height < NCHS standard	842	7–12 y	9.49 (1.72)	50	ONS vs. usual diet	365	Low
Han, 2011	USA	CDGM	20	7–11 y	9.30 (1.33)	100	ONS vs. usual diet	90, 180, 365	High
Sheng, 2014	China	WHP < 25th percentile, picky eater	142	2.5–5 y	3.71 (0.70)	49	ONS-DC vs. DC	30, 60, 90, 120	High
Lebenthal, 2014 Yackobovitch, 2016 Rawat, 2018	Israel	HAP and WAP ≤ 10th percentile; WAP ≤ HAP	200	3–9 y	5.50 (1.50)	75	ONS vs. low-caloric, low-protein control	90, 180	High
O’Reilly, 2015	Ireland	Undernourished	67	2–10 y	4.80 (2.00)	NR	ONS-DC vs. DC	16, 42	Low
Cervo, 2017	Philippines	3 ≤ WAZ < −2 or WAZ < −3	80	3–5 y	4.19 (1.27)	37	ONS vs. usual diet	84 ^	Moderate
Ghosh (1), 2018 Ghosh (2), 2018	India	−2 ≤ WAZ < −1 Picky eater, URTI	255	2–6 y	3.67 (1.19)	63	ONS-DC vs. DC	10, 30, 60, 90	High
Khanna, 2019	India	3% < WHZ < 15% Picky eater	321	2–4 y	2.94 (0.54)	60	ONS-DC vs. DC	7, 30, 60, 90	High

* For weight and height, the anthropometric Z-scores and percentiles were based on WHO growth standard unless otherwise stated. ^Cervo study had 84 days of intervention period, which was pooled with those studies with 90 days of intervention. HAZ, height-for-age Z-score; WHZ, weight-for-height Z-score; m, months; ONS, oral nutritional supplements; WHP, weight-for-height Percentile; y, years; DC, dietary counselling; NCHS, National Center for Health Statistics; CDGM, Constitutional delay of growth and maturation; HAP, height-for-age percentile; WAP, weight-for-age percentile; NR, not reported; WAZ, weight-for-age Z-score.; URTI, upper-respiratory tract infection.

## Data Availability

Not applicable.

## Supplementary Materials

The following are available online at https://www.mdpi.com/article/10.3390/nu13093036/s1; Figure S1: Risk of bias for selected studies; Figure S2: Meta-analysis results on the difference in weight (kg) gain between intervention and control after 7–10 days of intervention; Figure S3: Meta-analysis results on the difference in mean change in weight parameters between intervention and control based on a subgroup analysis of RCTs comparing ONS + DC versus DC alone with repeated measures at 30, 60, and 90 days of intervention; Figure S4: Meta-analysis results on the difference in mean change in height parameters between intervention and control based on a subgroup analysis of RCTs comparing ONS + DC versus DC alone with repeated measures at 30, 60, and 90 days of ONS intervention; Figure S5: Meta-analysis results on the difference in mean change in other anthropometric measures between intervention and control after 90 days of intervention; Figure S6: Meta-analysis results on the difference in mean change in total energy intake between intervention and control after 60 and 90 days of intervention; Figure S7: Funnel plots for the difference in mean change in weight and height parameters between intervention and control based on the longest follow-up time point; Table S1: Detailed search strategy for ProQuest Dialogue (PQD); Table S2: Percent difference in height gain between intervention and control.
