# Supplementary material for: Effect of Oral Nutritional Supplementation on Growth in Children with Undernutrition: A Systematic Review and Meta-Analysis
Source: Nutrients. 2021 Aug 30;13(9):3036. doi: 10.3390/nu13093036 (PMC8468927; doi:10.3390/nu13093036)
Supplement: Supplementary file 1 [file nutrients-13-03036-s001.zip › nutrients-1293417-supplementary.pdf]

Supplemental Table S1. Detailed search strategy for ProQuest Dialogue (PQD)

PQD Search Strategy

Databases: BIOSIS Previews®, CAB ABSTRACTS, Embase®, EMCare®, FSTA®, MEDLINE®, ProQuest Dissertations and Theses Professional, ToxFile®

TI,AB("oral nutrition supplement*" or "oral nutritional supplement*" or "supplementary oral nutrition" or "supplemented oral nutrition" or "supplementary enteral nutrition" or "supplemented enteral nutrition" or "supplemental enteral nutrition" or ((oral or orally) and ("enteral formula*" or "enteric formula*")) or "polymeric formula*" or "polymeric diet*" or "oral nutritional intervention*" or "sip feed*" or "nutrition* drink" or "nutrition* drinks" or "milk drink" or "milk drinks" or "fortified milk*" or "enriched milk*" or "fortified beverage*" or "liquid nutrition* supplement*" or "oral enteral nutrition*" or "oral enteric nutrition*" or "oral enteral feeding*" or "protein energy supplement*" or "protein-energy supplement*" or "protein and energy supplement*" or "protein calorie supplement*" or "protein-calorie supplement*" or "protein and calorie supplement*" or "oral nutrition*" or ((oral or orally) n/10 nutrition* n/10 supplement*) or ("oral supplement*" and (nutrition* or nutrient*)) or ((oral or orally) and ("enteral nutrition*" or “enteral supplement*” or "enteral feeding*")) or “nutrition supplement*” or “nutritional supplement*” or “growing-up milk” or “growing-up formula*” or “grow-up milk” or “grow-up formula*” or “toddler milk” or “toddler formula*” or “toddler’s milk” or “toddler’s formula*” or “toddlers’ milk” or “toddlers’ formula*” or “follow-up milk” or “follow-up formula*” or “follow-on milk” or “follow-on formula*”) OR EMB.EXACT ("artificial milk") OR MESH.EXACT("Milk Substitutes" or "Food, Formulated")

S1 AND TI,AB(child or schoolchild or children or schoolchildren or "school child*" or kid or kids or toddler* or adoles* or preadolescen* or pre-adolescen* or preteen* or teen* or boy* or girl* or minors or underage* or "under ag*" or juvenile* or youth* or kindergar* or puber* or pubescen* or prepubescent* or prepuberty* or pediatric* or paediatric* or peadiatric* or schools or "nursery school*" or preschool* or "pre school*" or "primary school*" or "secondary school*" or "elementary school*" or "high school*" or highschool* or "school age" or schoolage or "school age*" or schoolage*)

TI,AB(stunted or stunting or underweight or “under-weight” or wasting or wasted or thinness or “failure to thrive” or “faltering weight” or “weight faltering” or “low weight” or “low height” or "growth retardation" or "growth faltering" or "growth failure" or “failure to grow” or "delayed growth*" or “restricted growth*” or “suboptimal growth*” or “sub-optimal growth*” or “poor growth*” or “poor physical growth” or “insufficient weight gain*” or “slow weight gain*” or “catch-up growth*” or “catchup growth*” or malnutrition or malnourishment or malnourished or “mal-nourished” or “under-nourished” or undernourishment or undernutrition or “under-nutrition” or undernourished or “nutrition* risk*” or “risk of nutrition” or "picky eating" or "picky eater*" or "fussy eating" or "fussy eater*" or “selective eating” or “selective eater*” or “restrictive food intake disorder*” or "avoidant food intake disorder*" or “food neophobia” or “feeding disorder*” or “infantile anorexia”)

TI,AB(growth* and (weigh* or height or heights or length or lengths or anthropometr* or “body mass index” or BMI or “skinfold thickness*” or “arm circumference*” or “muscle circumference*” or “head circumference*” or “chest circumference*” or “muscle area*” or “body size*” or “body composition*”)) OR EMB.EXACT.EXPLODE(“body weight” or “body height” or “arm circumference*” or “body mass” or “body fat” or “body size” or “weight height ratio” or “head circumference” or “chest circumference” or “body growth” or “growth rate and growth regulation” or “postnatal growth”) OR MESH.EXACT.EXPLODE(“Body Weights and Measures” or “body size” or “body weight” or “body height” or “body weight changes” or “Thinness” or “Body Mass Index” or “Skinfold Thickness” or “Body Composition”)

S2 AND (S3 OR S4)

S5 NOT TI,AB(cat or cats or feline or dog or dogs or canine* or rat or rats or mouse or mice or hamster* or gerbil* or rodent* or rabbit* or bunn* or fly or insect* or cattle or calves or Holstein* or heifer* or horse or sheep or (fish* not “fish oil”) or avian or murine or swine or pig or pigs or piglet or piglets or goose or geese or monkey or "cell culture" or “cell line”)

MESH.EXACT.EXPLODE(“Adult” not “Child”) or EMB.EXACT.EXPLODE(“adult” not “child”)
S6 NOT S7
S8 NOT TI((infant or infants or neonat* or newborn or newborns or prenatal or “pre-natal” or perinatal or “peri-natal” or postnatal or “post-natal” or preemie* or preterm* or adult or adults or women or men or elderly or geriatric or geriatrics) not (child or children))
S9 NOT TI(vitamin PRE/2 supplement* or “calcium supplement*” or “zinc supplement*” or “iron supplement*” or ((“parenteral nutrition*” or “parenteral alimentation” or tube or tubes) not (enteral or enteric or oral or orally)))
TI,AB(HIV or neoplasm or neoplasms or neoplastic or neoplasia or tumour or tumours or tumor or tumors or oncology or cancer or *carcinoma* or chemotherapy or chemotherapeutic or “inflammatory bowel disease*” or “Crohn’s disease” or “Crohn disease” or “celiac disease” or “cystic fibrosis” or “liver disease” or “cirrhosis” or "colitis" or gastroenteritis or "kidney disease" or "renal disease" or "renal failure*" or "kidney failure*" or *dialysis or “sickle-cell disease” or “sickle-cell an*mia” or “short bowel syndrome”)
S10 NOT S11
S10 AND S11
IF,SU("oral nutrition supplement*" or "oral nutritional supplement*" or "supplementary oral nutrition" or "supplemented oral nutrition" or "supplementary enteral nutrition" or "supplemented enteral nutrition" or "supplemental enteral nutrition" or ((oral or orally) and ("enteral formula*" or "enteric formula*")) or "polymeric formula*" or "polymeric diet*" or "oral nutritional intervention*" or "sip feed*" or "nutrition* drink" or "nutrition* drinks" or "milk drink" or "milk drinks" or "fortified milk*" or "enriched milk*" or "fortified beverage*" or "liquid nutrition* supplement*" or "oral enteral nutrition*" or "oral enteric nutrition*" or "oral enteral feeding*" or "protein energy supplement*" or "protein-energy supplement*" or "protein and energy supplement*" or "protein calorie supplement*" or "protein-calorie supplement*" or "protein and calorie supplement*" or "oral nutrition*" or ((oral or orally) n/10 nutrition* n/10 supplement*) or ("oral supplement*" and (nutrition* or nutrient*)) or ((oral or orally) and ("enteral nutrition*" or “enteral supplement*” or "enteral feeding*")) or “nutrition supplement*” or “nutritional supplement*” or “growing-up milk” or “growing-up formula*” or “grow-up milk” or “grow-up formula*” or “toddler milk” or “toddler formula*” or “toddler’s milk” or “toddler’s formula*” or “toddlers’ milk” or “toddlers’ formula*” or “follow-up milk” or “follow-up formula*” or “follow-on milk” or “follow-on formula*”) OR EMB.EXACT ("artificial milk") OR MESH.EXACT("Milk Substitutes" or "Food, Formulated")
S14 AND IF,SU(child or schoolchild or children or schoolchildren or "school child*" or kid or kids or toddler* or adoles* or preadolescen* or pre-adolescen* or preteen* or teen* or boy* or girl* or minors or underage* or "under ag*" or juvenile* or youth* or kindergar* or puber* or pubescen* or prepubescent* or prepuberty* or pediatric* or paediatric* or peadiatric* or schools or "nursery school*" or preschool* or "pre school*" or "primary school*" or "secondary school*" or "elementary school*" or "high school*" or highschool* or "school age" or schoolage or "school age*" or schoolage*)
IF,SU(stunted or stunting or underweight or “under-weight” or wasting or wasted or thinness or “failure to thrive” or “faltering weight” or “weight faltering” or “low weight” or “low height” or "growth retardation" or "growth faltering" or "growth failure" or “failure to grow” or "delayed growth*" or “restricted growth*” or “suboptimal growth*” or “sub-optimal growth*” or “poor growth*” or “poor physical growth” or “insufficient weight gain*” or “slow weight gain*” or “catch-up growth*” or “catchup growth*” or malnutrition or malnourishment or malnourished or “mal-nourished” or “under-nourished” or undernourishment or undernutrition or “under-nutrition” or undernourished or “nutrition* risk*” or “risk of nutrition” or "picky eating" or "picky eater*" or "fussy eating" or "fussy eater*" or “selective eating” or “selective eater*” or “restrictive food intake disorder*” or "avoidant food intake disorder*" or “food neophobia” or “feeding disorder*” or “infantile anorexia”)
IF,SU(growth* and (weigh* or height or heights or length or lengths or anthropometr* or “body mass index” or BMI or “skinfold thickness*” or “arm circumference*” or “muscle circumference*” or “head circumference*” or “chest circumference*” or “muscle area*” or “body size*” or “body composition*”)) OR EMB.EXACT.EXPLODE(“body weight” or “body height” or “arm circumference*” or “body mass” or “body fat” or “body size” or “weight height ratio” or “head circumference” or “chest circumference” or “body growth”

or “growth rate and growth regulation” or “postnatal growth”) OR MESH.EXACT.EXPLODE(“Body Weights and Measures” or “body size” or “body weight” or “body height” or “body weight changes” or “Thinness” or “Body Mass Index” or “Skinfold Thickness” or “Body Composition”)
S15 AND (S16 OR S17)
S18 NOT TI,AB,IF,SU(cat or cats or feline or dog or dogs or canine* or rat or rats or mouse or mice or hamster* or gerbil* or rodent* or rabbit* or bunn* or fly or insect* or cattle or calves or Holstein* or heifer* or horse or sheep or (fish* not “fish oil”) or avian or murine or swine or pig or pigs or piglet or piglets or goose or geese or monkey or "cell culture" or “cell line”)
MESH.EXACT.EXPLODE(“Adult” not “Child”) or EMB.EXACT.EXPLODE(“adult” not “child”)
S19 NOT S20
S21 NOT TI((infant or infants or neonat* or newborn or newborns or prenatal or “pre-natal” or perinatal or “peri-natal” or postnatal or “post-natal” or preemie* or preterm* or adult or adults or women or men or elderly or geriatric or geriatrics) not (child or children))
S22 NOT TI(vitamin PRE/2 supplement* or “calcium supplement*” or “zinc supplement*” or “iron supplement*” or ((“parenteral nutrition*” or “parenteral alimentation” or tube or tubes) not (enteral or enteric or oral or orally)))
IF,SU(HIV or neoplasm or neoplasms or neoplastic or neoplasia or tumour or tumours or tumor or tumors or oncology or cancer or *carcinoma* or chemotherapy or chemotherapeutic or “inflammatory bowel disease*” or “Crohn’s disease” or “Crohn disease” or “celiac disease” or “cystic fibrosis” or “liver disease” or “cirrhosis” or "colitis" or gastroenteritis or "kidney disease" or "renal disease" or "renal failure*" or "kidney failure*" or *dialysis or “sickle-cell disease” or “sickle-cell an*mia” or “short bowel syndrome”)
S23 NOT S24
S23 AND S24

Supplemental Table S2. Percent difference in height gain between intervention and control.

		Intervention		Control		
Study	Intervention Days	N	Mean (SD) Height Gain (cm)	N	Mean (SD) Height Gain (cm)	% Increase in Height Gain
Alarcon (2003)	90	44	2.66 (1.45)	47	1.72 (1.12)	55%
Khanna (2019)	90	204	1.43 (1.86)	101	1.02 (1.47)	40%
Ghosh (2018)	90	119	1.33 (1.43)	123	0.98 (1.13)	36%
Sheng (2014)	90	69	2.04 (0.73)	62	2.17 (0.71)	-6%

Author, Year	Randomization bias	Selection Bias	Performance Bias	Detection Bias	Attrition Bias	Reporting Bias	Other Biases
Walker, 2011	+	?	–	+	+	+	+
Alarcon, 2003	+	?	?	–	+	+	+
Schrezenmeir, 2004	?	?	–	+	+	+	+
Han, 2011	?	?	–	–	+	+	+
Khanna, 2019	+	?	+	+	?	+	+
Lebenthal, 2014 and Yackobovitch, 2016	?	?	+	+	–	+	+
Sheng, 2014	+	+	+	+	–	+	+
O'Reilley, 2015	?	?	–	?	+	?	?
Cervo, 2017	?	+	–	?	–	+	+
Ghosh, 2018	+	+	–	?	+	+	+
Vijayalakshmi, 2008	?	?	–	?	+	+	+

Supplemental Figure S1. Risk of Bias for selected studies.

Study name	Sample size: Control	Sample size: Intervention	Days	Statistics for each study: Difference in means	Standard error	Lower limit	Upper limit	p-Value
Ghosh (2018)	125	123	10	0.060	0.033	-0.004	0.124	0.068
Khanna (2019)	106	210	7	0.070	0.030	0.011	0.129	0.021
Schrezenmeir (2004)	44	85	8	0.122	0.023	0.077	0.167	0.000
				0.089	0.021	0.049	0.130	0.000

Difference in means and 95% CI

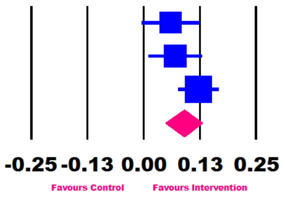

Supplemental Figure S2. Meta-analysis results on the difference in weight (kg) gain between intervention and control after 7-10 days of intervention. The forest plot shows the mean difference (squares) and 95% confidence intervals (CIs) (horizontal bars) for intervention vs control. The values were combined using a meta-analysis to obtain a pooled estimate of the effect from all the included studies (diamond).

S3a

Study	Sample size: Intervention	Sample size: Control	Days	Difference in means	Standard error	Lower limit	Upper limit	p-Value
Alarcon (2003)	44	43	30	0.230	0.085	0.064	0.396	0.007
Ghosh (2018)	121	124	30	0.240	0.044	0.154	0.326	0.000
Khanna (2019)	207	103	30	0.170	0.052	0.069	0.271	0.001
Sheng (2014)	74	67	30	0.126	0.068	-0.007	0.259	0.062
				0.198	0.028	0.143	0.253	0.000
Alarcon (2003)	44	45	60	0.540	0.105	0.333	0.747	0.000
Ghosh (2018)	120	124	60	0.330	0.050	0.233	0.427	0.000
Khanna (2019)	207	101	60	0.230	0.075	0.082	0.378	0.002
Sheng (2014)	70	64	60	0.184	0.088	0.011	0.357	0.037
				0.311	0.063	0.188	0.434	0.000
Alarcon (2003)	44	47	90	0.740	0.113	0.518	0.962	0.000
Ghosh (2018)	119	123	90	0.390	0.055	0.283	0.497	0.000
Khanna (2019)	204	101	90	0.390	0.098	0.199	0.581	0.000
Sheng (2014)	69	62	90	0.273	0.101	0.075	0.471	0.007
				0.438	0.083	0.275	0.601	0.000

Difference in means and 95% CI

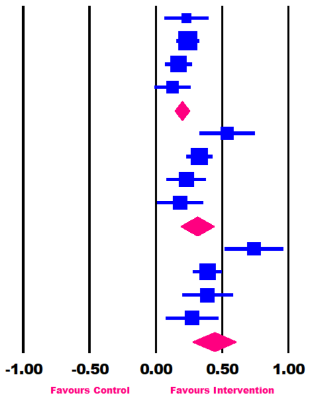

S3b

Study name	Sample size: Intervention	Sample size: Control	Days	Difference in means	Standard error	Lower limit	Upper limit	p-Value
Alarcon (2003)	44	43	30	0.089	0.096	-0.099	0.276	0.353
Ghosh (2018)	121	124	30	0.140	0.028	0.084	0.196	0.000
Khanna (2019)	207	103	30	0.110	0.038	0.036	0.184	0.004
Sheng (2014)	74	67	30	0.071	0.091	-0.108	0.250	0.437
				0.124	0.021	0.082	0.166	0.000
Alarcon (2003)	44	45	60	0.212	0.094	0.028	0.396	0.024
Ghosh (2018)	120	124	60	0.200	0.032	0.138	0.262	0.000
Khanna (2019)	207	101	60	0.140	0.051	0.041	0.239	0.006
Sheng (2014)	68	64	60	0.106	0.095	-0.080	0.292	0.265
				0.180	0.025	0.131	0.229	0.000
Alarcon (2003)	44	47	90	0.319	0.095	0.133	0.504	0.001
Ghosh (2018)	119	123	90	0.240	0.034	0.174	0.306	0.000
Khanna (2019)	204	101	90	0.240	0.061	0.120	0.360	0.000
Sheng (2014)	66	61	90	0.168	0.098	-0.024	0.360	0.087
				0.241	0.027	0.188	0.294	0.000

Difference in means and 95% CI

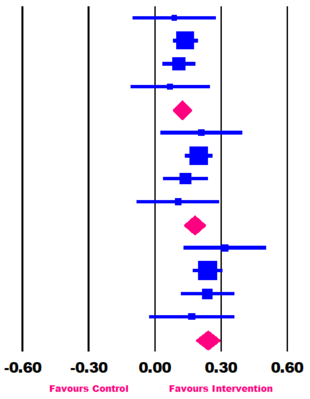

S3c

Study name	Sample size: Intervention	Sample size: Control	Days	Difference in means	Standard error	Lower limit	Upper limit	p-Value
Alarcon (2003)	44	43	30	2.480	1.097	0.331	4.629	0.024
Ghosh (2018)	121	124	30	2.700	0.553	1.616	3.784	0.000
Khanna (2019)	207	103	30	1.800	0.939	-0.040	3.640	0.055
Sheng (2014)	74	67	30	1.433	1.882	-2.256	5.122	0.447
				2.417	0.426	1.582	3.251	0.000
Alarcon (2003)	44	45	60	5.930	1.556	2.881	8.979	0.000
Ghosh (2018)	120	124	60	3.740	0.653	2.460	5.020	0.000
Khanna (2019)	207	101	60	2.840	1.340	0.215	5.465	0.034
Sheng (2014)	68	64	60	2.216	2.041	-1.785	6.217	0.278
				3.751	0.530	2.711	4.790	0.000
Alarcon (2003)	44	47	90	8.030	1.722	4.656	11.404	0.000
Ghosh (2018)	119	123	90	4.640	0.713	3.242	6.038	0.000
Khanna (2019)	204	101	90	5.350	1.702	2.014	8.686	0.002
Sheng (2014)	66	61	90	3.641	2.136	-0.544	7.827	0.088
				5.210	0.765	3.710	6.710	0.000

Difference in means and 95% CI

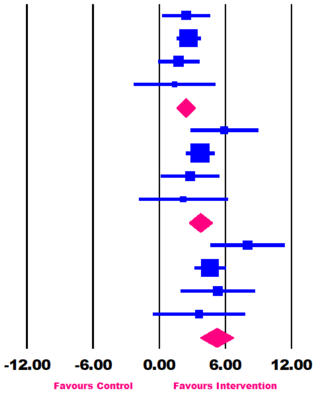

S3d

Study name	Sample size: Intervention	Sample size: Control	Days	Difference in means	Standard error	Lower limit	Upper limit	p-Value
Alarcon (2003)	44	43	30	0.119	0.073	-0.024	0.261	0.102
Ghosh (2018)	98	99	30	0.230	0.049	0.134	0.326	0.000
Khanna (2019)	207	103	30	0.130	0.058	0.016	0.244	0.025
Sheng (2014)	74	67	30	0.114	0.066	-0.016	0.244	0.085
				0.161	0.030	0.102	0.220	0.000
Alarcon (2003)	44	45	60	0.257	0.086	0.088	0.427	0.003
Ghosh (2018)	95	98	60	0.260	0.052	0.157	0.363	0.000
Khanna (2019)	207	101	60	0.190	0.068	0.056	0.324	0.005
Sheng (2014)	68	64	60	0.149	0.072	0.008	0.290	0.038
				0.219	0.033	0.154	0.284	0.000
Alarcon (2003)	44	47	90	0.372	0.083	0.210	0.535	0.000
Ghosh (2018)	93	96	90	0.270	0.061	0.150	0.390	0.000
Khanna (2019)	204	101	90	0.270	0.084	0.105	0.435	0.001
Sheng (2014)	66	61	90	0.227	0.073	0.084	0.370	0.002
				0.279	0.037	0.207	0.351	0.000

Difference in means and 95% CI

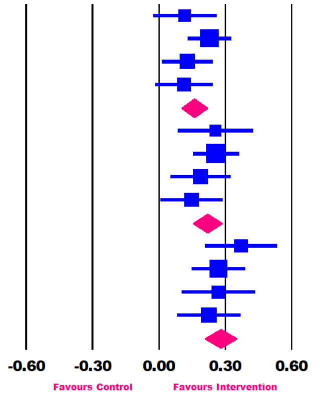

S3e

Study name	Sample size: Intervention	Sample size: Control	Days	Difference in means	Standard error	Lower limit	Upper limit	p-Value
Alarcon(2003)	44	43	30	3.300	1.944	-0.511	7.111	0.090
Ghosh (2018)	98	99	30	5.500	1.161	3.224	7.776	0.000
Khanna(2019)	207	103	30	3.810	1.445	0.977	6.643	0.008
Sheng (2014)	74	67	30	1.849	1.071	-0.250	3.949	0.084
				3.606	0.904	1.835	5.376	0.000
Alarcon(2003)	44	45	60	9.080	2.732	3.726	14.434	0.001
Ghosh (2018)	95	98	60	6.820	1.204	4.460	9.180	0.000
Khanna(2019)	207	101	60	5.510	1.926	1.735	9.285	0.004
Sheng (2014)	68	64	60	2.575	1.231	0.163	4.987	0.036
				5.570	1.368	2.889	8.251	0.000
Alarcon(2003)	44	47	90	11.750	2.749	6.363	17.137	0.000
Ghosh (2018)	93	96	90	8.020	1.547	4.988	11.052	0.000
Khanna(2019)	204	101	90	8.510	2.428	3.751	13.269	0.000
Sheng (2014)	66	61	90	3.848	1.205	1.485	6.210	0.001
				7.519	1.703	4.181	10.857	0.000

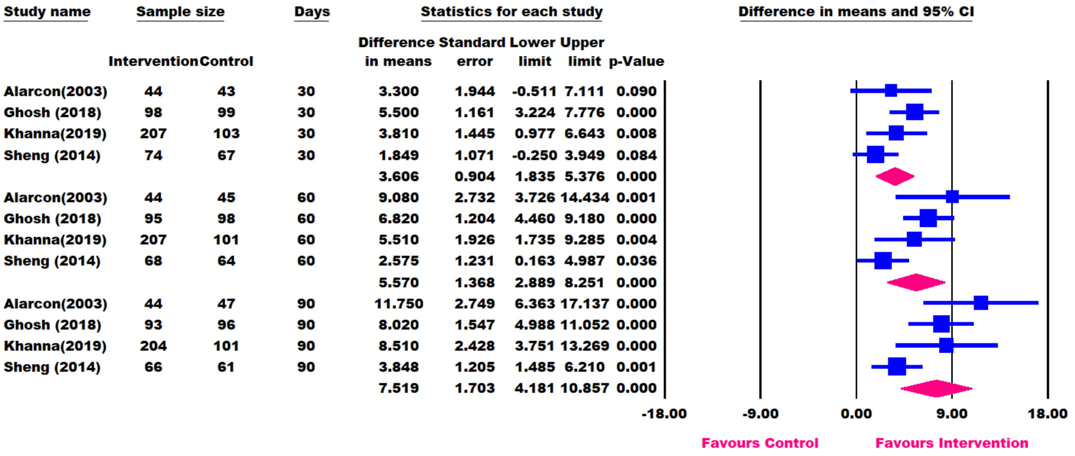

Supplemental Figure S3. Meta-analysis results on the difference in mean change in weight parameters between intervention and control based on a subgroup analysis of RCTs comparing ONS+DC versus DC alone with repeated measures at 30, 60, and 90 days of intervention. **S3a.** Weight (kg), **S3b.** Weight-for-age Z-score (WAZ), **S3c.** Weight-for-age percentile (WAP), **S3d.** Weight-for-height Z-score (WHZ), S**3e.** Weight-for-height percentile (WHP). The forest plot shows the mean difference (squares) and 95% confidence intervals (CIs) (horizontal bars) for intervention vs control. The values were combined using a meta-analysis to obtain a pooled estimate of the effect from all the included studies (diamond).

S4a

Study	Sample size		Group by Days	Statistics for each study				
	Intervention	Control		Difference in means	Standard error	Lower limit	Upper limit	p-Value
Alarcon (2003)	44	43	30	0.480	0.215	0.059	0.901	0.025
Ghosh (2018)	121	124	30	0.020	0.084	-0.145	0.185	0.812
Khanna (2019)	207	103	30	0.180	0.127	-0.068	0.428	0.155
Sheng (2014)	74	67	30	-0.139	0.105	-0.345	0.067	0.186
				0.081	0.102	-0.118	0.281	0.423
Alarcon (2003)	44	45	60	0.660	0.255	0.159	1.161	0.010
Ghosh (2018)	120	124	60	0.150	0.124	-0.092	0.392	0.225
Khanna (2019)	207	101	60	0.150	0.161	-0.166	0.466	0.352
Sheng (2014)	70	64	60	-0.147	0.115	-0.372	0.078	0.200
				0.144	0.136	-0.123	0.411	0.290
Alarcon (2003)	44	47	90	0.940	0.271	0.410	1.470	0.001
Ghosh (2018)	119	123	90	0.350	0.165	0.026	0.674	0.034
Khanna (2019)	204	101	90	0.410	0.212	-0.005	0.825	0.053
Sheng (2014)	69	62	90	-0.138	0.126	-0.385	0.109	0.274
				0.350	0.215	-0.072	0.772	0.104

Difference in means and 95% CI

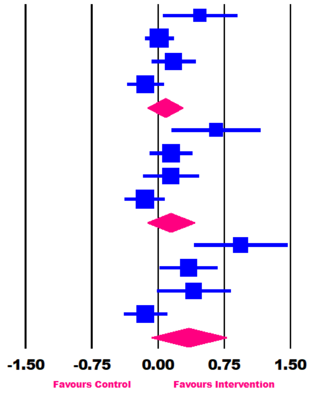

S4b

Study name	Sample size		Days	Statistics for each study				
	Intervention	Control		Difference in means	Standard error	Lower limit	Upper limit	p-Value
Alarcon (2003)	44	43	30	0.038	0.126	-0.208	0.285	0.760
Ghosh (2018)	121	124	30	0.000	0.022	-0.043	0.043	1.000
Khanna (2019)	207	103	30	0.050	0.034	-0.017	0.117	0.145
Sheng (2014)	74	67	30	0.002	0.142	-0.277	0.281	0.989
				0.015	0.018	-0.021	0.050	0.415
Alarcon (2003)	44	45	60	0.114	0.125	-0.132	0.359	0.363
Ghosh (2018)	120	124	60	0.040	0.032	-0.023	0.103	0.212
Khanna (2019)	207	101	60	0.040	0.044	-0.047	0.127	0.367
Sheng (2014)	68	64	60	0.033	0.147	-0.256	0.322	0.823
				0.043	0.025	-0.006	0.092	0.088
Alarcon (2003)	44	47	90	0.125	0.126	-0.121	0.372	0.318
Ghosh (2018)	119	123	90	0.080	0.043	-0.003	0.163	0.060
Khanna (2019)	204	101	90	0.100	0.057	-0.013	0.213	0.082
Sheng (2014)	66	61	90	0.048	0.149	-0.245	0.341	0.748
				0.088	0.032	0.025	0.151	0.006

Difference in means and 95% CI

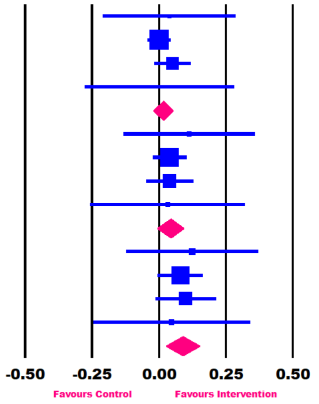

S4c

Study	Sample size: Intervention	Sample size: Control	Days	Difference in means	Standard error	Lower limit	Upper limit	p-Value
Alarcon (2003)	44	43	30	1.850	1.216	-0.534	4.234	0.128
Ghosh (2018)	121	124	30	1.020	0.452	0.134	1.906	0.024
Khanna (2019)	207	103	30	0.850	0.497	-0.124	1.824	0.087
Sheng (2014)	74	67	30	0.020	4.924	-9.631	9.671	0.997
				1.003	0.322	0.372	1.633	0.002
Alarcon (2003)	44	45	60	3.170	1.147	0.922	5.418	0.006
Ghosh (2018)	120	124	60	2.220	0.606	1.033	3.407	0.000
Khanna (2019)	207	101	60	1.270	0.769	-0.237	2.777	0.099
Sheng (2014)	68	64	60	1.090	5.114	-8.933	11.113	0.831
				2.042	0.438	1.184	2.900	0.000
Alarcon (2003)	44	47	90	5.240	1.317	2.659	7.821	0.000
Ghosh (2018)	119	123	90	3.700	0.854	2.025	5.375	0.000
Khanna (2019)	204	101	90	2.640	1.230	0.230	5.050	0.032
Sheng (2014)	66	61	90	1.590	5.218	-8.636	11.816	0.761
				3.741	0.615	2.536	4.947	0.000

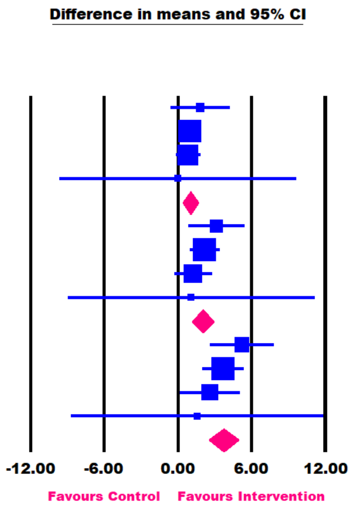

Supplemental Figure S4. Meta-analysis results on the difference in mean change in height parameters between intervention and control based on a subgroup analysis of RCTs comparing ONS+DC versus DC alone with repeated measures at 30, 60, and 90 days of ONS intervention. **S4a.** Height (cm), **S4b.** Height-for-age Z-score (HAZ), **S4c.** Height-for-age percentile (HAP). The forest plot shows the mean difference (squares) and 95% confidence intervals (CIs) (horizontal bars) for intervention vs control. The values were combined using a meta-analysis to obtain a pooled estimate of the effect from all the included studies (diamond).

S5a

Study	Subgroup	Sample size: Intervention	Sample size: Control	Days	Difference in means	Standard error	Lower limit	Upper limit	p-Value
Cervo (2017)	SU	20	20	90	0.200	0.242	-0.275	0.675	0.409
Cervo (2017)	UW	20	20	90	0.930	0.223	0.493	1.367	0.000
Ghosh (2018)	All	119	123	90	0.190	0.058	0.076	0.304	0.001
Khanna (2019)	All	204	101	90	0.150	0.060	0.033	0.267	0.012
					0.279	0.102	0.080	0.479	0.006

Difference in means and 95% CI

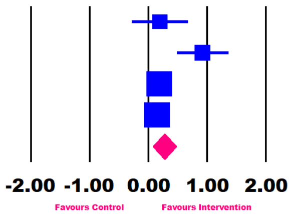

S5b

Study name	Subgroup	Sample size: Intervention	Sample size: Control	Days	Difference in means	Standard error	Lower limit	Upper limit	p-Value
Cervo (2017)	SU	20	20	90	0.590	0.219	0.161	1.019	0.007
Cervo (2017)	UW	20	20	90	1.240	0.252	0.745	1.735	0.000
Ghosh (2018)	All	119	123	90	0.350	0.061	0.230	0.470	0.000
Han (2011)	All	10	10	90	-0.200	0.247	-0.684	0.284	0.418
Khanna (2019)	All	204	101	90	0.320	0.110	0.104	0.536	0.004
					0.434	0.143	0.154	0.714	0.002

Difference in means and 95% CI

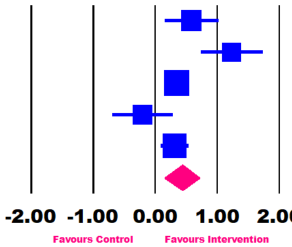

S5c

Study name	Sample size: Intervention	Sample size: Control	Time point	Difference in means	Standard error	Lower limit	Upper limit	p-Value
Ghosh (2018)	119	123	90	0.270	0.054	0.165	0.375	0.000
Han (2011)	10	10	90	-0.100	0.134	-0.363	0.163	0.456
Khanna (2019)	204	101	90	0.260	0.089	0.085	0.435	0.004
				0.177	0.093	-0.006	0.360	0.058

Difference in means and 95% CI

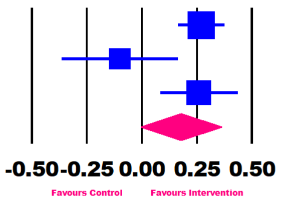

Supplemental Figure S5: Meta-analysis results on the difference in mean change in other anthropometric measures between intervention and control after 90 days of intervention. **S5a.** Mid-arm circumference (MAC), **S5b.** Body mass index (BMI), **S5c.** BMI for age Z-score (BMIAZ). The forest plot shows the mean difference (squares) and 95% confidence intervals

(CIs) (horizontal bars) for intervention vs control. The values were combined using a meta-analysis to obtain a pooled estimate of the effect from all the included studies (diamond).

Study	Subgroup	Sample size		Days	Statistics for each study				
		Intervention	Control		Difference in means	Standard error	Lower limit	Upper limit	p-Value
Ghosh (2018)	All	120	124	60	11.4	39.2	-65.5	88.3	0.771
Khanna (2019)	All	206	101	60	119.8	44.1	33.4	206.1	0.007
Sheng (2014)	All	71	63	60	179.7	35.2	110.7	248.6	0.000
					104.4	51.5	3.4	205.3	0.043
Cervo (2017)	SU	20	20	90	538.0	61.1	418.2	657.8	0.000
Cervo (2017)	UW	20	20	90	607.0	57.4	494.4	719.6	0.000
Ghosh (2018)	All	119	123	90	71.8	37.9	-2.5	146.1	0.058
Khanna (2019)	All	204	101	90	160.1	45.6	70.6	249.6	0.000
					341.3	132.1	82.3	600.3	0.010

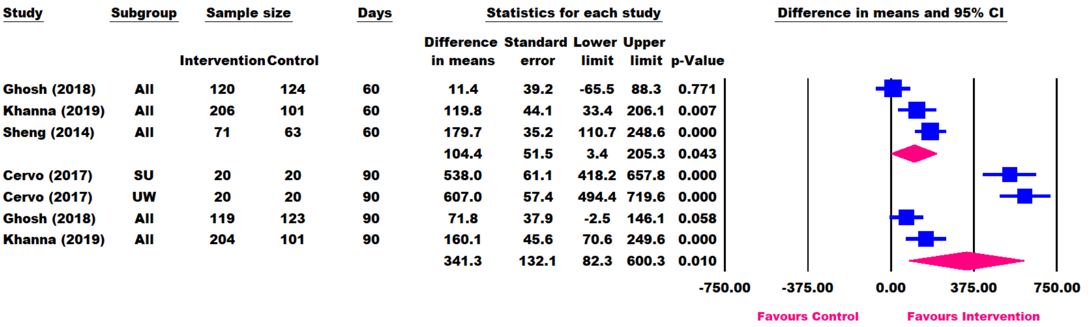

Supplemental Figure S6. Meta-analysis results on the difference in mean change in total energy intake between intervention and control after 60 and 90 days of intervention. SU: severely underweight; UW: underweight. The forest plot shows the mean difference (squares) and 95% confidence intervals (CIs) (horizontal bars) for intervention vs control. The values were combined using a meta-analysis to obtain a pooled estimate of the effect from all the included studies (diamond).

S7a

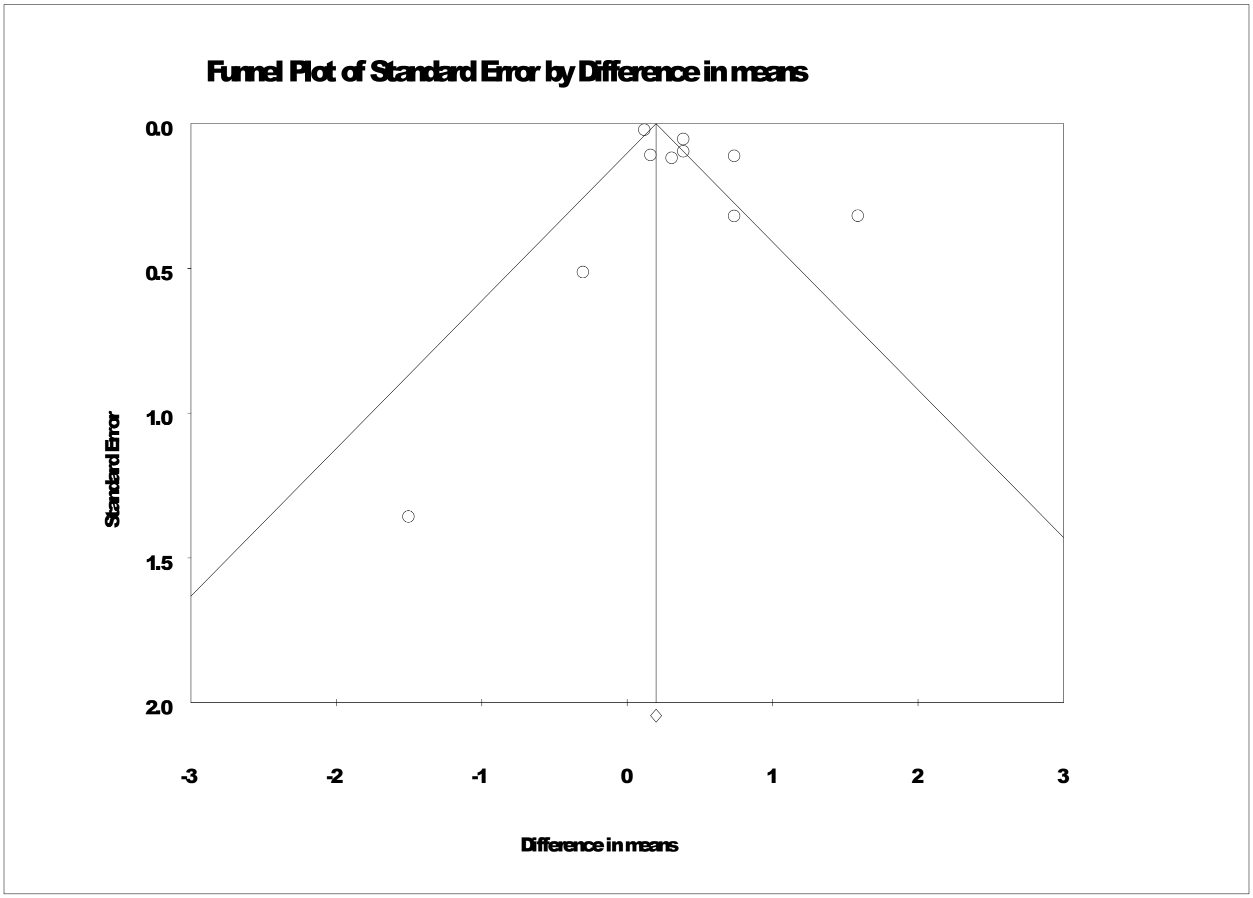

Funnel Plot of Standard Error by Difference in means
0.0
0.5
1.0
1.5
2.0
Standard Error
-3
-2
-1
0
1
2
3
Difference in means

S7b

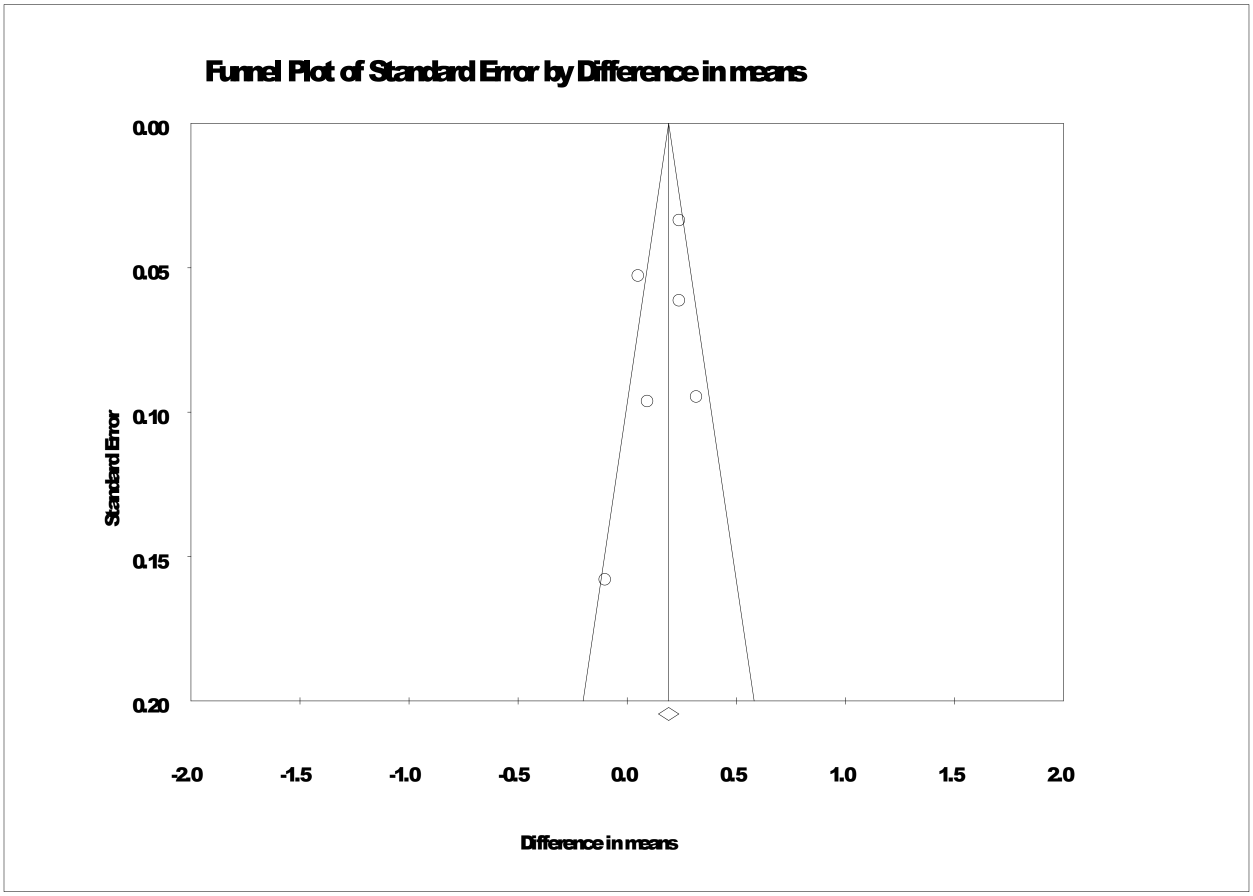

Funnel Plot of Standard Error by Difference in means
0.00
0.05
0.10
0.15
0.20
Standard Error
-2.0
-1.5
-1.0
-0.5
0.0
0.5
1.0
1.5
2.0
Difference in means

S7c

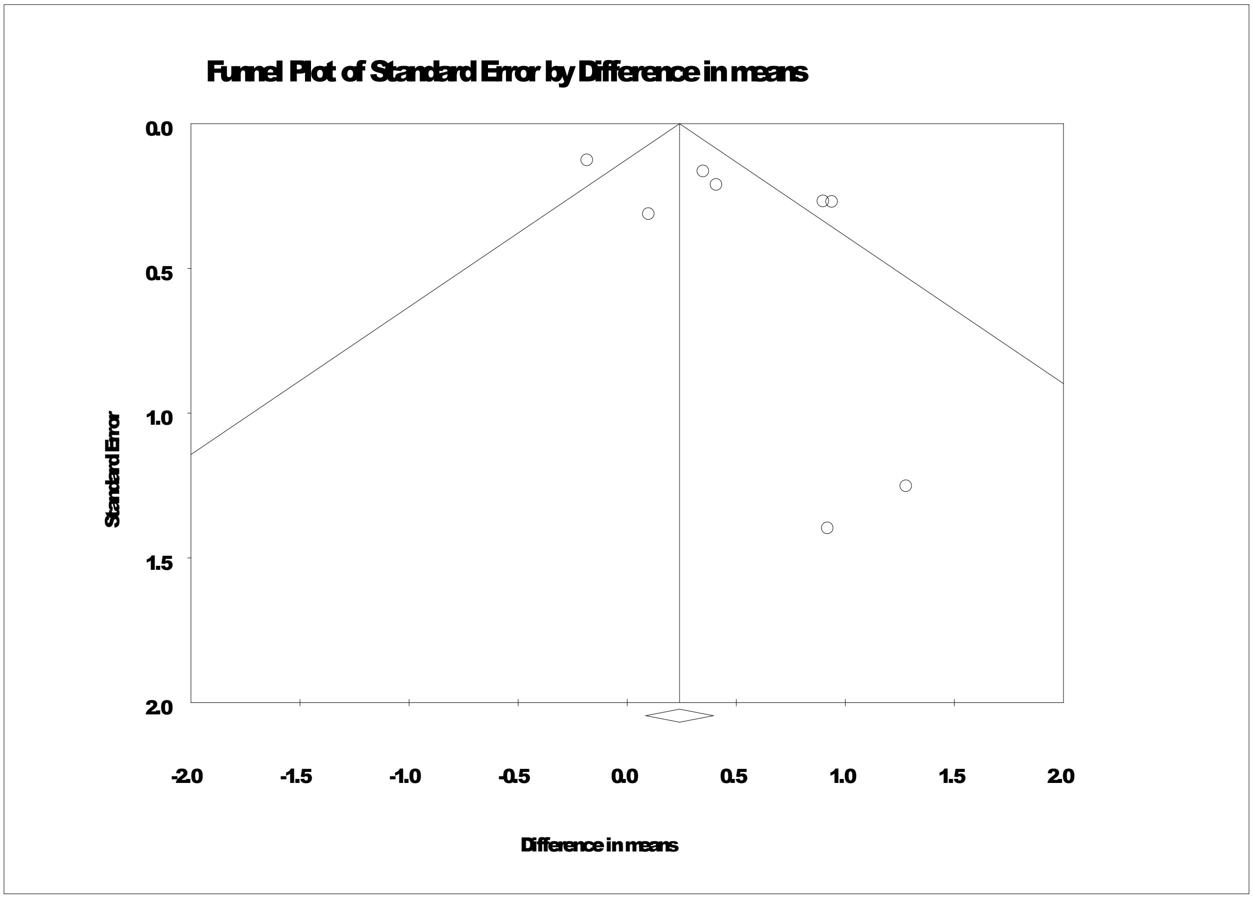

Funnel Plot of Standard Error by Difference in means
Standard Error
0.0
0.5
1.0
1.5
2.0
-2.0
-1.5
-1.0
-0.5
0.0
0.5
1.0
1.5
2.0
Difference in means

S7d

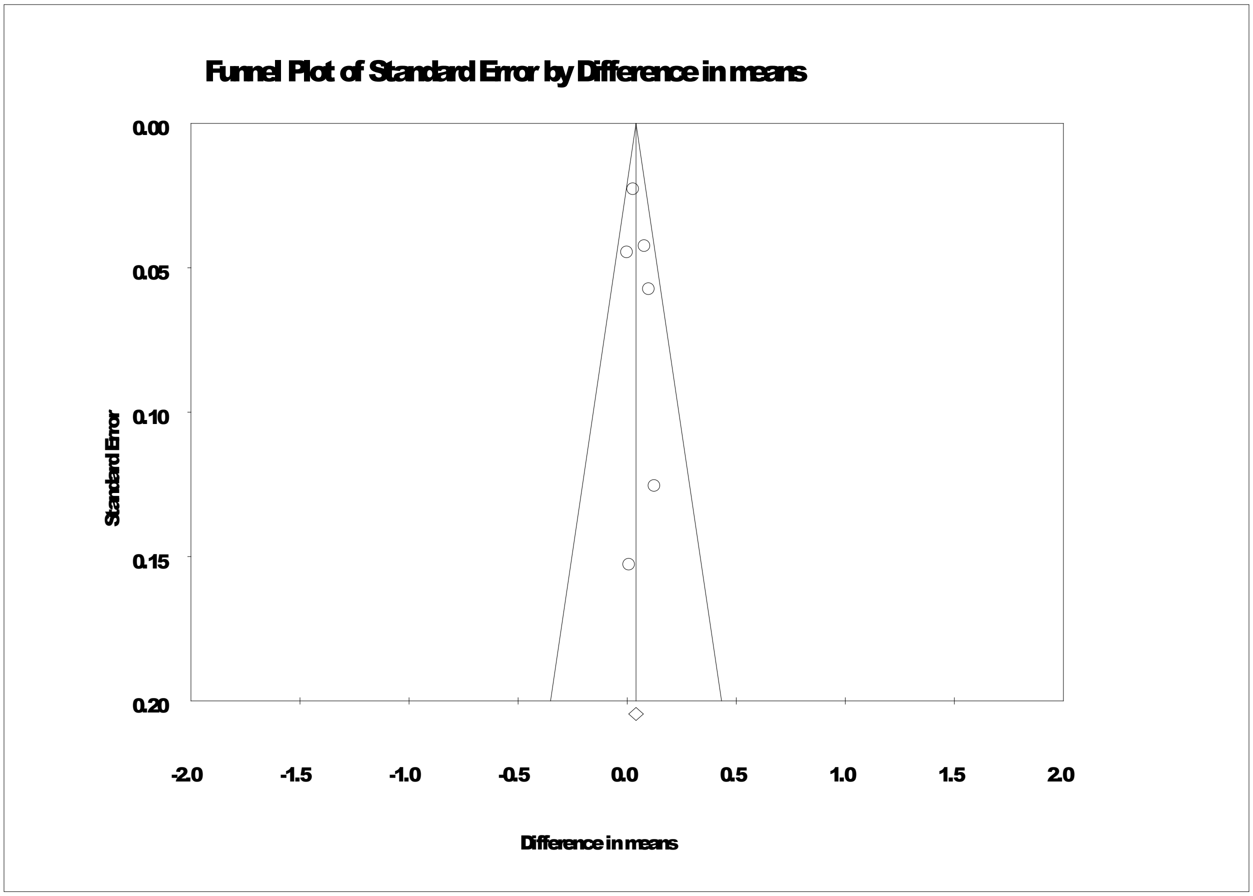

Supplemental Figure S7. Funnel plots for the difference in mean change in weight and height parameters between intervention and control based on the longest follow-up time point. **S7a.** Weight (kg), **S7b.** Weight-for-age Z-score (WAZ), **S7c.** Height (cm), **S7d.** Height-for-age Z-score (HAZ).
